# Reactive Molecular Dynamics Study on Soot Formation Mechanism in Iron-Doped Ethylene Combustion

**DOI:** 10.3390/molecules31213889

**Published:** 2026-10-28

**Authors:** Yang Wang, Yiju Zhao, Junjie Lu, Yuan Zhu, Zhenhui Zhang, Lumin Chen, Mingyan Gu

**Affiliations:** 1 Maanshan General Hospital of Ranger-Duree Healthcare, Ma’anshan 243000, China; 2 Engineering Research Center of Biofilm Water Purification and Utilization, Technology of Ministry of Education, Anhui University of Technology, Ma’anshan 243032, China; 3 School of Mechanical and Electrical Engineering, Tongling University, Tongling 244061, China

**Keywords:** soot, PAHs, iron-hydrocarbon co-combustion, ReaxFF MD, Fe powder

## Abstract

Iron (Fe) powder is a promising zero-carbon energy carrier, and its co-combustion with hydrocarbon fuels offers a transitional route toward decarbonized power generation. Fe has nevertheless been experimentally found to aggravate early soot nucleation and growth in flames while reducing engine-out soot emissions, and the atomic-scale mechanism by which Fe regulates polycyclic aromatic hydrocarbons (PAHs) and soot formation remains unresolved. To address this gap, reactive molecular dynamics (ReaxFF MD) was employed to investigate PAHs and soot formation during the combustion of ethylene (C_2_H_4_) doped with Fe, across three reaction temperatures (2000, 2500, and 3000 K) and three Fe loading levels (0, 6, and 12 Fe atoms, corresponding to Fe/C atomic ratios of 0, 2.5%, and 5.0%). The CHOFe-2012.ff reactive force field was selected for the Fe-containing simulations because it reproduces the experimentally validated Fe-free ethylene combustion benchmarks most closely among the force fields compared, while also describing Fe-C and Fe-O interactions. At 20 ns and 3000 K, the largest soot cluster detected contains 191 carbon atoms with a C/H ratio of 7.07, favoring the formation of highly graphitized mature soot. In contrast, under 2000 K, the corresponding largest cluster possesses only 77 carbon atoms and exhibits a C/H ratio of 3.35, yielding only loosely structured young soot. It should be noted that these descriptors characterize the most evolved cluster identified in each simulation rather than the entire soot population. Within the tested conditions, Fe doping is found to promote soot formation: both the carbon-atom number and the C/H ratio of the largest soot cluster increase markedly, and this effect strengthens with increasing Fe loading. Notably, the O_2_ level varied by less than 2.2% across the Fe-loading series, and these descriptors refer to the largest detected cluster; within the present simulation framework, the promotion is therefore attributed predominantly to the chemical effect of Fe doping. The reaction path analysis reveals that Fe atoms form Fe-containing small carbon nuclei by combining with C_2_H_4_, dehydrogenation and addition reactions, and then gradually develop into mature soot particles through coalescence, surface growth and graphitization, indicating that Fe participates in the nucleation stage of soot formation under the tested conditions. This study provides a molecular-level understanding of the microscopic mechanism of Fe–soot interactions, offering mechanistic insight at the molecular scale that may inform the optimization of Fe-hydrocarbon dual-fuel systems and the mitigation of Fe-induced soot formation.

## 1. Introduction

Soot particles produced by the incomplete combustion of hydrocarbon fuels are a major source of atmospheric fine particulate matter and pose a sustained risk to human health [1]. Iron (Fe) powder is a promising energy carrier, referred to here as “zero-carbon” in the sense that its combustion stage oxidizes the metal directly to Fe oxides without producing CO_2_, unlike carbon-containing hydrocarbon fuels; it further benefits from high volumetric energy density, abundant reserves, and the recyclability of its combustion products via hydrogen-based regeneration [2,3]. Although it still faces engineering challenges, including Fe-particle ignition difficulty [4], insufficient oxidation [5], and the high cost of product regeneration [6], co-firing Fe powder with hydrocarbon fuels in combustion devices has been proposed as a highly viable transitional approach [7], since the hydrocarbon fuel provides reliable ignition and high energy density while the Fe contributes its low-carbon characteristics, with the two fuels complementing each other in combustion performance. Underlying these practical issues is a fundamental, yet unresolved question—namely, how the presence of Fe regulates soot formation during hydrocarbon–Fe co-combustion—and addressing this question is of direct scientific and engineering significance.

Regarding experimental investigations on soot regulation by Fe-based additives, it should be recognized that these studies employ Fe in different chemical states-including elemental (metallic) Fe powder, organometallic ferrocene, and Fe-oxide nanoparticles -which can lead to different soot-regulating behaviors and therefore complicate direct comparisons among studies. Most in-engine studies report that Fe-based additives reduce particulate emissions through catalytic effects; for example, Resitoglu et al. [8] summarized that metal-based additives, including Fe-based ones, reduce particulate emissions catalytically, Almanzalawy et al. [9] verified that Fe_3_O_4_ nanoparticles mitigate smoke emissions and shorten combustion duration in diesel-biodiesel blends, and a further study using Fe-doped ceria nanoparticles [10] reported reductions in NOx and CO emissions. By contrast, flame-level studies employing ferrocene (an organic Fe compound) show that Fe promotes early soot nucleation and particle growth [11,12,13]. Because of these differences in Fe chemical state, the available results are not directly comparable, and an atomic-scale mechanism common to these systems remains to be clarified. The present study focuses on elemental Fe in its atomic (gas-phase) form, representing the limiting case of fully vaporized metallic Fe in the flame, which is the form most directly relevant to Fe-powder co-combustion.

Soot formation covers the complete evolutionary chain from gas-phase small molecules to polycyclic aromatic hydrocarbons (PAHs), and ultimately to soot nanoparticles. It involves multiphase and multiscale physicochemical processes including gas-phase chemical reactions, nucleation, coalescence, surface growth, and oxidation. Although conventional macroscopic experimental approaches can obtain macroscopic parameters such as soot concentration, particle-size distribution and micromorphology, they cannot directly observe the atomic-scale fine evolutionary details during soot nucleation and early-stage growth. Miller et al. [14] employed TEM and EDS analyses in Fe-doped diesel combustion and revealed that when the Fe-to-carbon ratio in the combustion chamber exceeded 0.013, homogeneously nucleated metal nanoparticles began to form and agglomerate, with both their number and size increasing with higher Fe loading. Zhou et al. [11] experimentally and numerically investigated the soot particle size distribution in Fe-doped premixed ethylene (C_2_H_4_) flames, finding that Fe predominantly existed in the atomic state rather than in oxide form within the flame, and that as soot particles coagulated with one another, the Fe atoms incorporated in soot gradually aggregated into larger Fe cores. Further studies [12] demonstrated that the promoting effect of ferrocene on soot size growth is primarily achieved through enhanced inter-particle coagulation rather than surface growth. This mechanistic understanding is consistent with the conclusions of Mao et al. [13], who reported that Fe atoms promote the activation and heterogeneous nucleation of PAHs in the early stage of combustion. Engine tests confirmed that Fe-containing additives can reduce total soot emissions, but basic flame experiments found that Fe will aggravate early soot nucleation and particle growth. The two types of conclusions seem to be contradictory, but in fact, there are differences in the role of Fe in different combustion stages and regions, and the relevant microscopic mechanisms still need to be clarified.

Macroscopic experimental methods can provide macroscopic information such as soot concentration, particle size distribution, and morphology, and certain spectroscopic or indirect techniques (e.g., laser-induced fluorescence/incandescence and mass spectrometry) can probe intermediate species [15]. However, these approaches generally do not resolve the bond-level, atomistic evolution of the transient nucleation and early-growth species in as much detail as reactive molecular dynamics simulation, which is the motivation for employing ReaxFF MD in this work. The reactive molecular dynamics (ReaxFF MD) method uses a bond-level dependent potential function to dynamically track the breaking and formation of chemical bonds during the simulation process, which is particularly suitable for studying the pyrolysis, combustion, oxidation and polymerization of hydrocarbon fuels at high temperatures. Recent studies have successfully used ReaxFF MD to reveal the auxiliary mechanism of CO_2_ and H_2_O in the oxidation of PAHs [16], the evolution of PAHs during the pyrolysis of different furan fuels [17], and the formation mechanism of nitrogen-containing PAHs [18], thereby demonstrating the effectiveness and reliability of this method in the study of soot formation. In addition, a recently published ReaxFF MD study achieved rapid prediction of the PAH oxidation process [19], further expanding the applicable boundary of the method.

In this study, ReaxFF MD was used to explore the characteristics of soot formation during the combustion of typical gaseous fuel C_2_H_4_ doped with Fe powder. Although ReaxFF molecular dynamics has been applied to a range of Fe/C/H/O systems—including Fe oxidation, Fe-catalyzed hydrocarbon chemistry, and the interaction of Fe-containing nanoparticles with soot precursors—direct ReaxFF MD investigations of the complete PAH-to-soot formation pathway in C_2_H_4_–Fe powder co-combustion remain scarce; to our knowledge, the directly relevant studies (approximately 4–5) focus predominantly on Fe–soot/PAH interactions [11,13] rather than on the full formation pathway addressed here. The following testable hypotheses were formulated: (H1) Fe doping promotes PAH activation and accelerates soot nucleation and growth relative to pure-ethylene combustion at the same temperature; (H2) increasing Fe loading (Fe/C atomic ratio) enhances the growth and maturity of soot; (H3) elevating the reaction temperature accelerates soot nucleation, growth, and graphitization; and (H4) Fe atoms initiate the nucleation stage by combining with C_2_H_4_ to form Fe-containing carbon cores, which subsequently coalesce into primary Fe-containing soot. By comparing the elemental composition, C/H ratio and microstructure of soot, and adopting a pure-ethylene control group to isolate the independent effect of Fe, this work examines PAH formation, soot nucleation, and growth across three Fe loadings (0, 6, and 12 Fe atoms, i.e., Fe/C atomic ratios of 0, 2.5%, and 5.0%) and three reaction temperatures (2000, 2500, and 3000 K), and reveals the micro-mechanism governing Fe-modulated soot evolution. This study elucidates the Fe–soot interaction mechanism at the molecular level, providing theoretical support for soot regulation and clean-combustion optimization of Fe-hydrocarbon composite fuels.

## 2. Results and Discussion

### 2.1. Comparison of Species from Pure C_2_H_4_ Combustion Under Different ReaxFF Reactive Force Fields and Selection of the Fe-Containing Force Field

Table 1 presents the peak concentrations of the major combustion species during pure C_2_H_4_ combustion simulated using the four different ReaxFF reactive force fields. It is found that the ten major combustion species identified by the four force fields remain consistent across all simulations, namely H_2_O, C_2_H_4_, O_2_, H_2_, C_2_H_2_, C_2_H_2_O, C_2_H_3_, CO_2_, CO and C_2_H_2_O_2_, indicating that all four reactive force fields exhibit reasonable accuracy in simulating the C_2_H_4_ combustion process. Specifically, C_2_H_4_ and O_2_, as the fuel and oxidizer, are continuously consumed during combustion. Therefore, their peak values obtained from all four force fields are equal to their initial values. For the remaining eight species, the peak values predicted by the experimentally validated CHO-2016.ff and HE2.ff force fields are also in close agreement with each other, as demonstrated in previous studies. Consequently, these two force fields can serve as reliable benchmarks for validating the Fe-containing force fields CHOFe-2012.ff [20] and CHOFe-2015.ff [21].

For CHOFe-2012.ff, the evolution of species during the simulation of pure C_2_H_4_ combustion reveals that the peak values of the four combustion species—C_2_H_2_O, C_2_H_3_, CO_2_, and C_2_H_2_O_2_—are lower than those obtained with CHO-2016.ff and HE2.ff. In contrast, the peak values of H_2_O and CO_2_ predicted by CHOFe-2015.ff are lower than those of CHO-2016.ff and HE2.ff, while the peak values of H_2_ and CO exhibit the opposite trend. Therefore, based on the peak concentrations of the major combustion species and their temporal evolution, CHOFe-2012.ff shows better agreement with the experimentally validated reference force fields (CHO-2016.ff and HE2.ff) in simulating pure C_2_H_4_ combustion than the alternative Fe-containing force field (CHOFe-2015.ff).

To strengthen the validation of the selected force field, we additionally quantified the deviations of the species peak concentrations predicted by CHOFe-2012.ff from the experimentally validated benchmark force fields (CHO-2016.ff and HE2.ff). The peak concentrations of the major combustion species (H_2_O, H_2_, CO, C_2_H_2_) agree within 12%, while the relatively larger deviations of the minor species (C_2_H_3_, C_2_H_2_O, CO_2_, C_2_H_2_O_2_) reflect their low absolute abundances and do not affect the conclusions. Furthermore, the dominance of H_2_O, CO, and H_2_ predicted by CHOFe-2012.ff is consistent with the well-established stoichiometric expectations for fuel-rich (φ = 4) C_2_H_4_ combustion. Regarding the Fe-containing chemistry, the Fe–C and Fe–O interaction parameters of CHOFe-2012.ff were validated in its original development study [20]. To further assess the force field against experimental observations, we note that the predicted major-product distribution for rich C_2_H_4_ combustion (φ = 4)—with H_2_O, CO, and H_2_ as the dominant products and CO_2_ as a minor product—is consistent with the well-established product distribution in fuel-rich hydrocarbon flames. The predicted CO/CO_2_ ratio of approximately 2:1 at 2500 K also falls within the range reported for rich ethylene combustion. Additionally, the qualitative finding that Fe promotes early PAH activation and soot nucleation is consistent with independent flame experiments by Zhou et al. [12] and Mao et al. [13]. Nevertheless, direct quantitative experimental validation of Fe/C/H/O combustion kinetics remains unavailable, and the force-field predictions should therefore be interpreted as relative indicators rather than absolute quantitative values.

**Figure 1:**
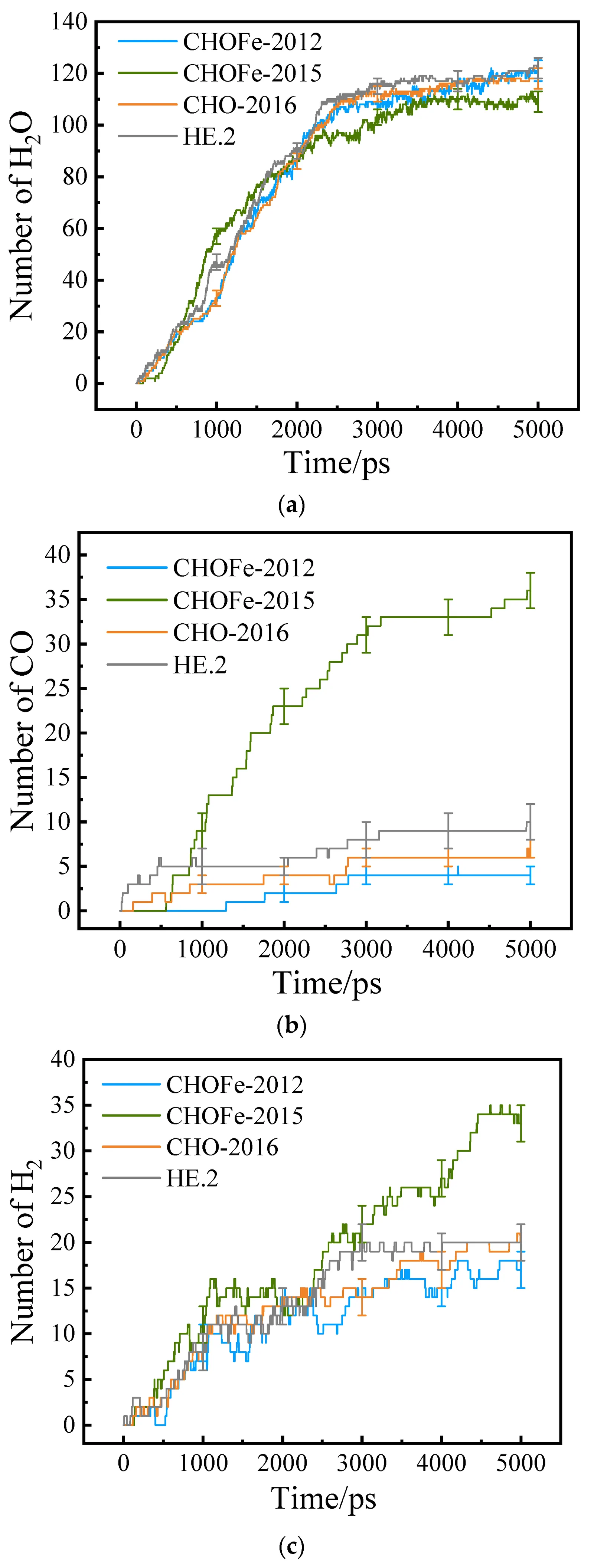
Temporal evolution of typical species during pure C_2_H_4_ combustion under different reactive force fields: (**a**) H_2_O, (**b**) CO, (**c**) H_2_. Error bars denote mean ± 1 SD over three independent simulations.

Given that H_2_O, CO, and H_2_ are the major products during the rich combustion of C_2_H_4_, Figure 1 further presents the temporal evolution of H_2_O, CO, and H_2_ within 5000 ps as simulated by the four different reactive force fields. It can be observed from Figure 1 that, on one hand, the evolution trends of these three products predicted by CHOFe-2012.ff and CHOFe-2015.ff are generally consistent with each other, further demonstrating the accuracy of both force fields in simulating C_2_H_4_ combustion. On the other hand, the evolution trends obtained with CHOFe-2012.ff are in agreement with those of CHO-2016.ff and HE2.ff, whereas CHOFe-2015.ff exhibits poorer agreement, particularly for CO and H_2_, where notable discrepancies are observed. Therefore, based on the combined analysis of Table 1 and Figure 1, CHOFe-2012.ff is selected as the reactive force field for the subsequent investigation of soot formation characteristics and mechanisms.

To further quantify the agreement between the force fields, the deviations between the CHOFe-2012.ff predictions and the experimentally validated benchmark force fields (CHO-2016.ff and HE2.ff) were evaluated at representative time points of the time series of the major products (H_2_O, CO, and H_2_). The resulting mean deviations remain small and are consistent with the peak-value agreement reported in Table 1, where the peak concentrations of the major species agree within 12%. Combined with the close temporal-profile agreement shown in Figure 1, these observations confirm that CHOFe-2012.ff reproduces the benchmark kinetics of the major products to a good approximation, supporting its selection for the Fe-containing simulations.

The CHOFe-2012.ff used in this paper was developed by Zou et al. [20] and applied to the Fe-containing C/H/O catalytic reaction system. The force field can capture the chemical interactions of Fe-C and Fe-O. The core scientific goal of this work is to reveal the qualitative reaction path of Fe-promoted soot nucleation, rather than to pursue high-precision quantitative prediction of the condensed phase properties of Fe oxides. In this paper, combined with Table 1 and Figure 1, the benchmark verification of the hydrocarbon-oxygen system is completed by an Fe-free combustion example. CHOFe-2012.ff was developed by Zou and van Duin [20] for Fe-containing C/H/O catalytic systems and has been used in a limited number of ReaxFF MD studies of Fe/C/H/O systems [20,22].

Accordingly, the conclusions drawn with CHOFe-2012.ff in this work should be read with a clear distinction between qualitative and quantitative claims. The qualitative conclusions—namely the overall reaction pathway by which Fe atoms combine with C_2_H_4_ to initiate carbon-core formation, the promoting role of Fe in the PAHs-nucleation stage, and the direction of the temperature- and Fe-loading-induced trends—are well supported by the simulation and are expected to be transferable across Fe/hydrocarbon co-combustion systems. In contrast, the quantitative parameters reported herein (e.g., the absolute carbon-atom number and C/H ratio of the largest soot cluster) should be regarded as relative indicators rather than high-precision predictions. Because all cases were simulated with the same force field, constant number of atoms and volume, and controlled temperature (NVT) ensemble, thermostat, and bond-order analysis cutoff, the comparative (relative) trends between different temperatures and Fe loadings are internally self-consistent and therefore meaningful, even though their absolute magnitudes cannot be directly compared with experiments. Several limitations of CHOFe-2012.ff for the Fe/C/H/O system should nevertheless be noted: (i) the force field was originally parameterized for Fe surface catalytic chemistry [20], and its description of Fe-O condensed phases is not intended to quantitatively reproduce bulk properties; (ii) the simulation temperatures (2000–3000 K) exceed typical practical combustion temperatures, so quantitative transferability to engine-relevant conditions is limited; and (iii) the soot-maturity thresholds (32 and 100 carbon atoms) adopted for classification are heuristic criteria chosen for consistent comparison rather than physically measured boundaries. Within these limits, the qualitative mechanisms identified here provide molecular-level insight into Fe-regulated soot formation.

### 2.2. Effects of Different Reaction Temperatures on Species and Soot Formation During C_2_H_4_ Combustion with Fe Addition

#### 2.2.1. Influence of Reaction Temperature on Soot Formation

**Figure 2:**
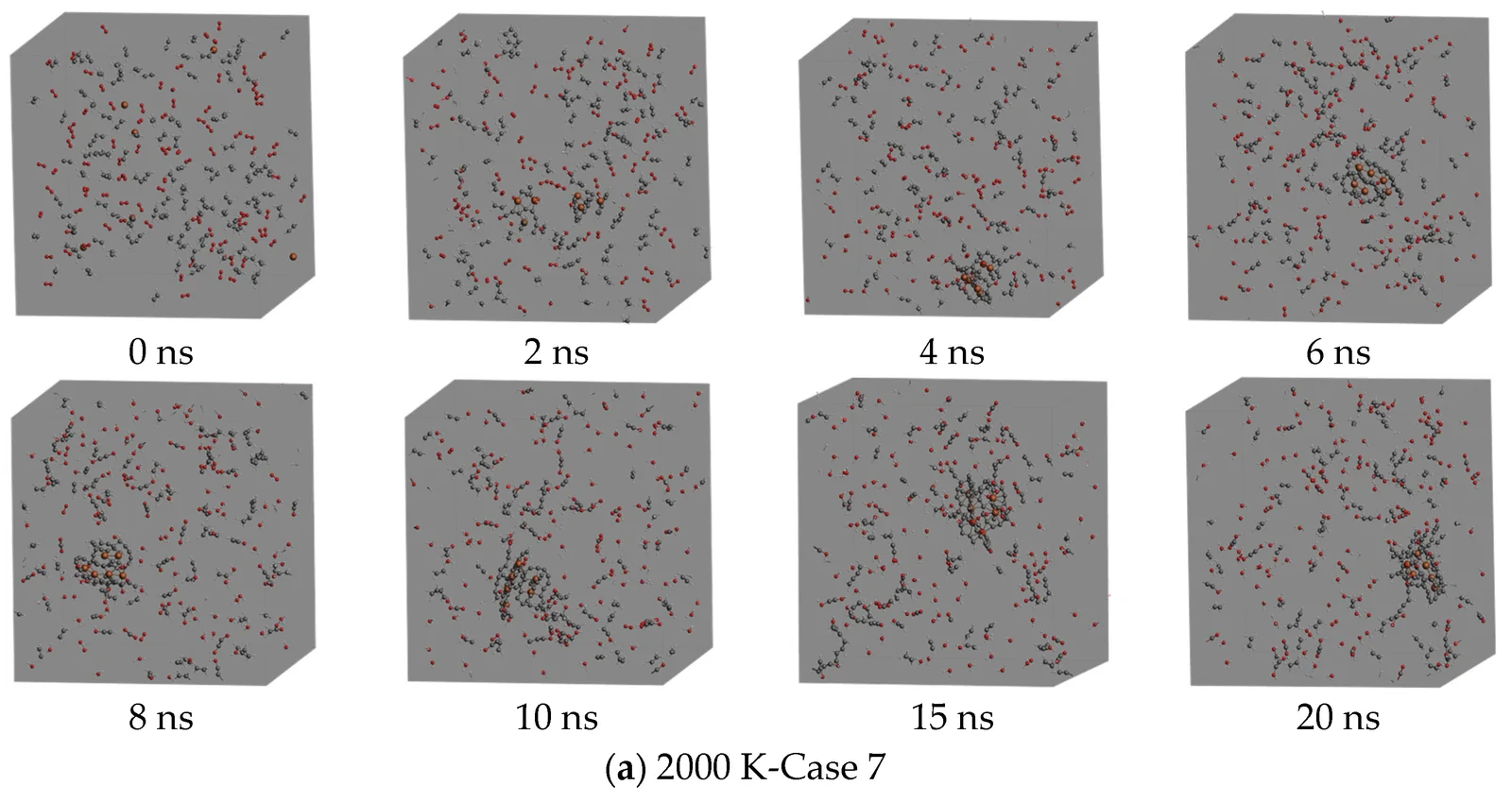

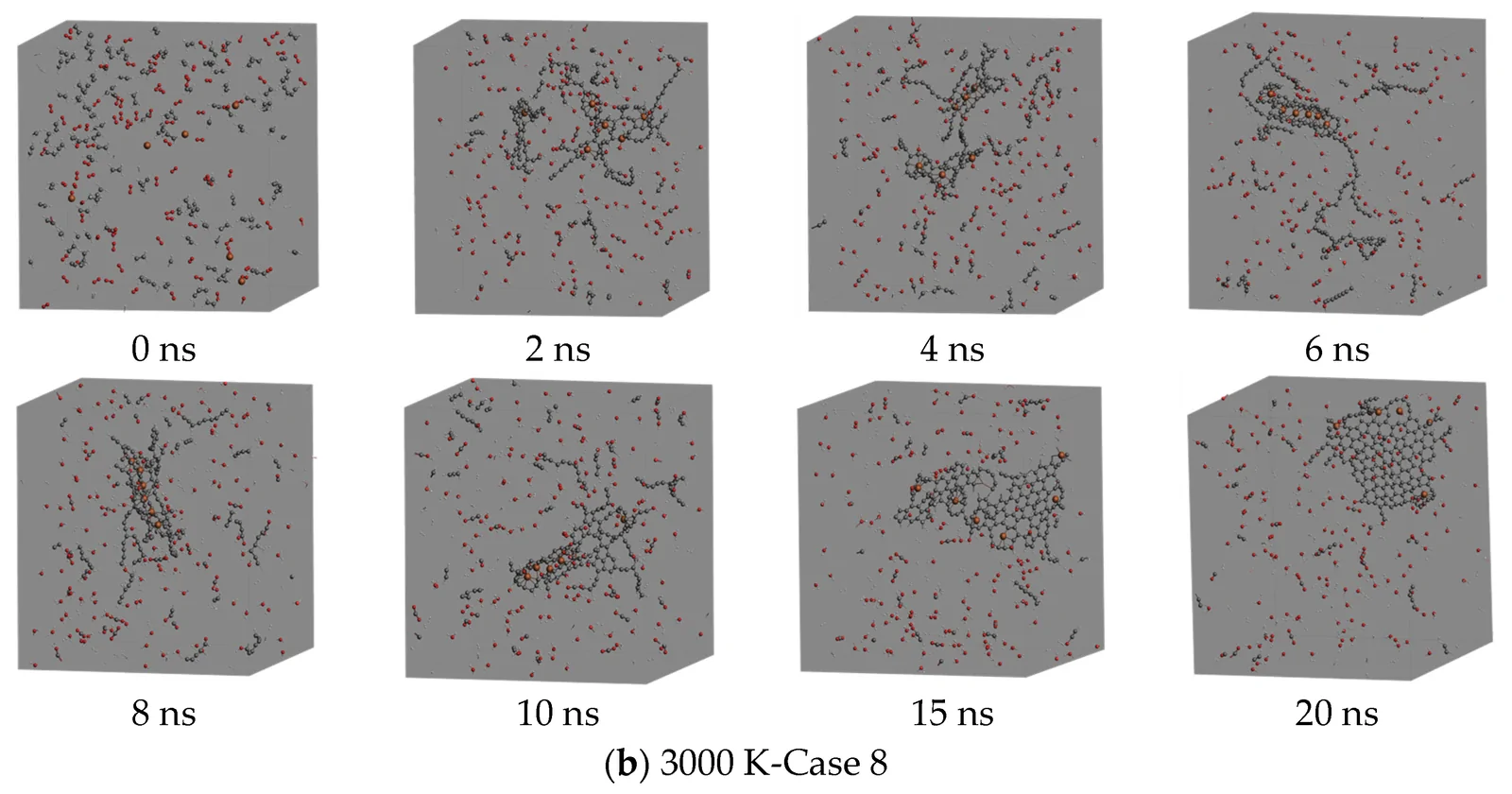
Effect of reaction temperature on soot formation during C_2_H_4_ combustion with Fe addition: (**a**) 2000 K; (**b**) 3000 K.

Figure 2a and Figure 2b illustrate the temporal evolution of PAHs and soot during Fe-doped ethylene combustion at 2000 K and 3000 K, respectively. To avoid subjective judgements relying solely on simulation snapshots, the total carbon atom number (N_C), together with the fractions of aromatic rings and aliphatic short-chain moieties, are adopted as quantitative criteria in this work to classify nascent (N_C < 32), young (32 ≤ N_C ≤ 100), and mature (N_C > 100) soot particles, following the classification criteria used in previous ReaxFF-based soot studies [23,24,25]. The exact mathematical definitions of these descriptors-including the aromaticity index and the aliphatic carbon fraction—are provided in Section 3. Furthermore, the aromaticity index and aliphatic carbon fraction are introduced as quantitative parameters for characterizing particle structures. As shown in Figure 2a, from the onset of the reaction to 2 ns, the fuel undergoes continuous pyrolysis, forming two Fe-containing PAHs, which are small “carbon cores” containing three Fe atoms. By 4 ns, these two Fe-containing PAHs further coagulate into one large nascent Fe-containing soot particle, characterized by a large “carbon core” containing six Fe atoms; however, the carbon content within this nascent soot particle remains quite limited at this stage. Through continuous surface growth, by 10 ns, a few five-membered and six-membered ring structures begin to form inside the particle. At 20 ns, the particle evolves into a young Fe-containing soot particle. Nevertheless, a considerable number of short carbon chains still exist within the ensemble at this point, indicating that the young Fe-containing soot particle still requires substantial time to mature into a fully developed Fe-containing soot particle. This is primarily attributable to the relatively low reaction temperature, which results in sluggish PAHs and soot formation rates.

Figure 2b presents the evolution of PAHs or soot during C_2_H_4_ combustion with Fe addition at 3000 K. From the onset of the reaction to 2 ns, it can be seen that clusters with more than 32 carbon atoms and a continuous covalent-bond network structure have emerged in this snapshot, indicating that the soot particles have completed the nucleation process. One large nascent Fe-containing soot particle containing five Fe atoms and one small nascent Fe-containing soot particle containing a single Fe atom have already formed. As the reaction proceeds to 4 ns, these two nascent soot particles coalesce and bond to form a young Fe-containing soot particle. With further reaction to 8 ns, the young Fe-containing soot particle undergoes continuous surface growth. By 10 ns, numerous ring structures emerge within the Fe-containing soot. At 15 ns, the carbon atoms within the soot are predominantly organized into five-membered and six-membered ring structures, and the particle has grown into a mature Fe-containing soot particle. During the final 5 ns of the simulation, the mature Fe-containing soot particle begins to undergo graphitization, adopting a more ordered structure, while the Fe atoms within the particle migrate toward the edges of the soot particle. The comparison between Figure 2a and Figure 2b shows that the production rate of Fe-containing soot particles is accelerated at a reaction temperature of 3000 K. The fuel pyrolysis and soot nucleation process have been completed in a simulation time of 2 ns, and the soot graphitization stage has entered within 20 ns. The Fe-containing soot particles at 2000 K temperature have not yet formed primary soot particles within 2 ns, and are still young Fe-containing soot particles at 20 ns. The carbon atoms inside the soot surround the Fe core and have not yet formed mature soot particles. It should be noted that, although Figure 2 presents the evolution of a representative trajectory for clarity, the key structural features described—the formation of Fe-containing carbon cores, the timing of soot nucleation, and the progression from nascent to mature soot—were observed consistently across all three independent NVT trajectories for each condition. This reproducibility is further corroborated by the population statistics and the reaction-frequency analysis reported in Section 2.3, indicating that the snapshot-based description is representative of the independent simulations rather than specific to a single trajectory.

**Figure 3:**
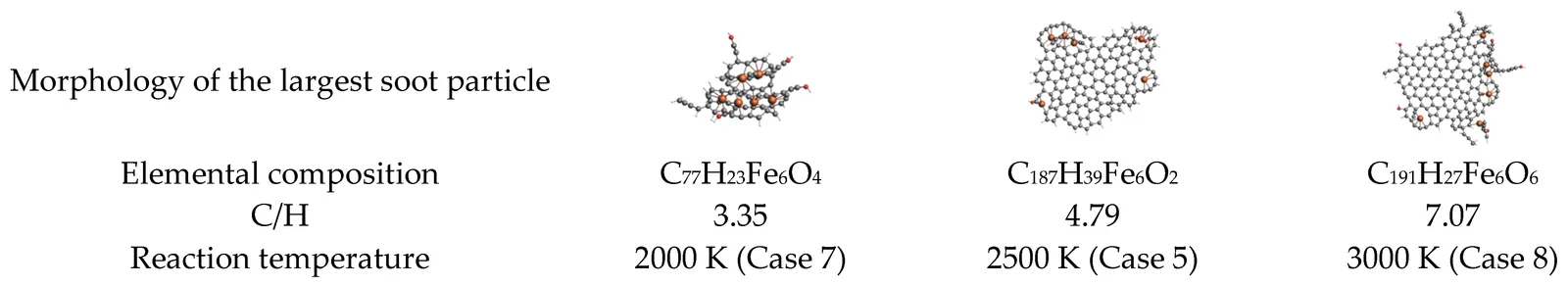
Morphology of the largest Fe-containing soot particles during C_2_H_4_ combustion at different reaction temperatures.

Figure 3 further illustrates the morphology and elemental composition of the largest soot particle formed from the onset of reaction to 20 ns under Fe-doped combustion conditions at three different reaction temperatures. The C/H ratio is used here as a soot-maturity indicator: soot maturation is characterized by progressive dehydrogenation and aromatization, which monotonically increase the carbon-to-hydrogen ratio as nascent aggregates evolve toward mature, more graphitized structures [26,27,28]. It should be noted that the analyses based on this largest particle can be used to compare the evolution behaviors of large soot clusters under different conditions, whereas the relevant conclusions apply only to the maximum soot cluster detected in the simulated system. This largest particle exhibits typical structural and compositional characteristics; those with fewer than 32 carbon atoms in chemical composition are defined as nascent soot, those with more than 100 carbon atoms as mature soot, and those falling between 32 and 100 as young soot. It can be found that the largest soot particles generated at 2000 K are still young Fe-containing soot particles based on Fe, which contain only 77 carbon atoms and C/H is only 3.35. As the reaction temperature increases to 2500 K, mature Fe-containing soot particles have been formed; the count of carbon atoms inside the soot increases to 187, and the C/H ratio also increases. Quantitatively, the normalized Fe-COM distance (d/R, where d is the Fe distance to the cluster center of mass and R is the carbon-core radius) increases from ~0.3–0.4 at 2–4 ns to ~0.8–0.9 at 20 ns, confirming that the Fe atoms migrate from the interior of the soot toward its surface during maturation. When the reaction temperature is further increased to 3000 K, although the count of carbon atoms in the soot did not increase further, the C/H increased from 4.79 to 7.07, indicating that the maturity of Fe-containing soot particles was improved. In summary, it can be found that the Fe-containing soot particles generated at 2000 K, 2500 K and 3000 K at the same time are young soot, mature soot and highly mature soot particles, respectively. The rise in reaction temperature can effectively promote the formation and growth of Fe-containing soot particles.

#### 2.2.2. The Influence of Reaction Temperature on the Evolution of Components

**Figure 4:**
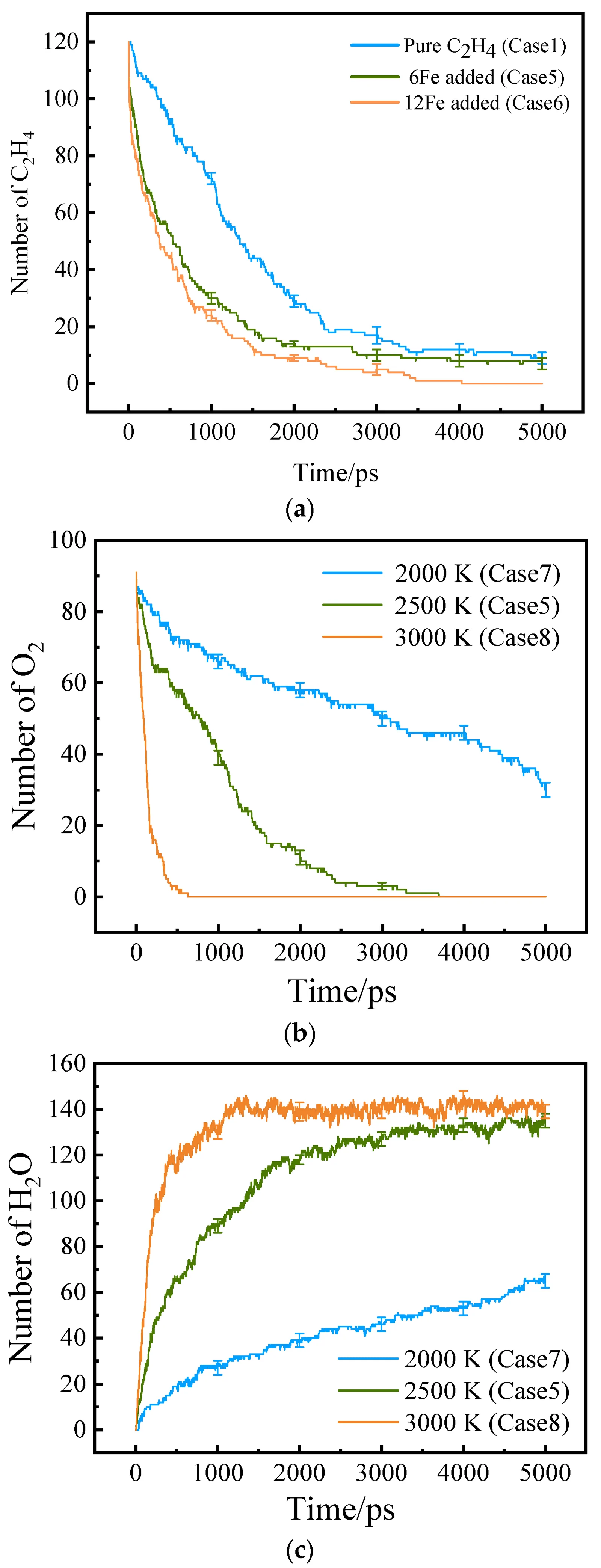

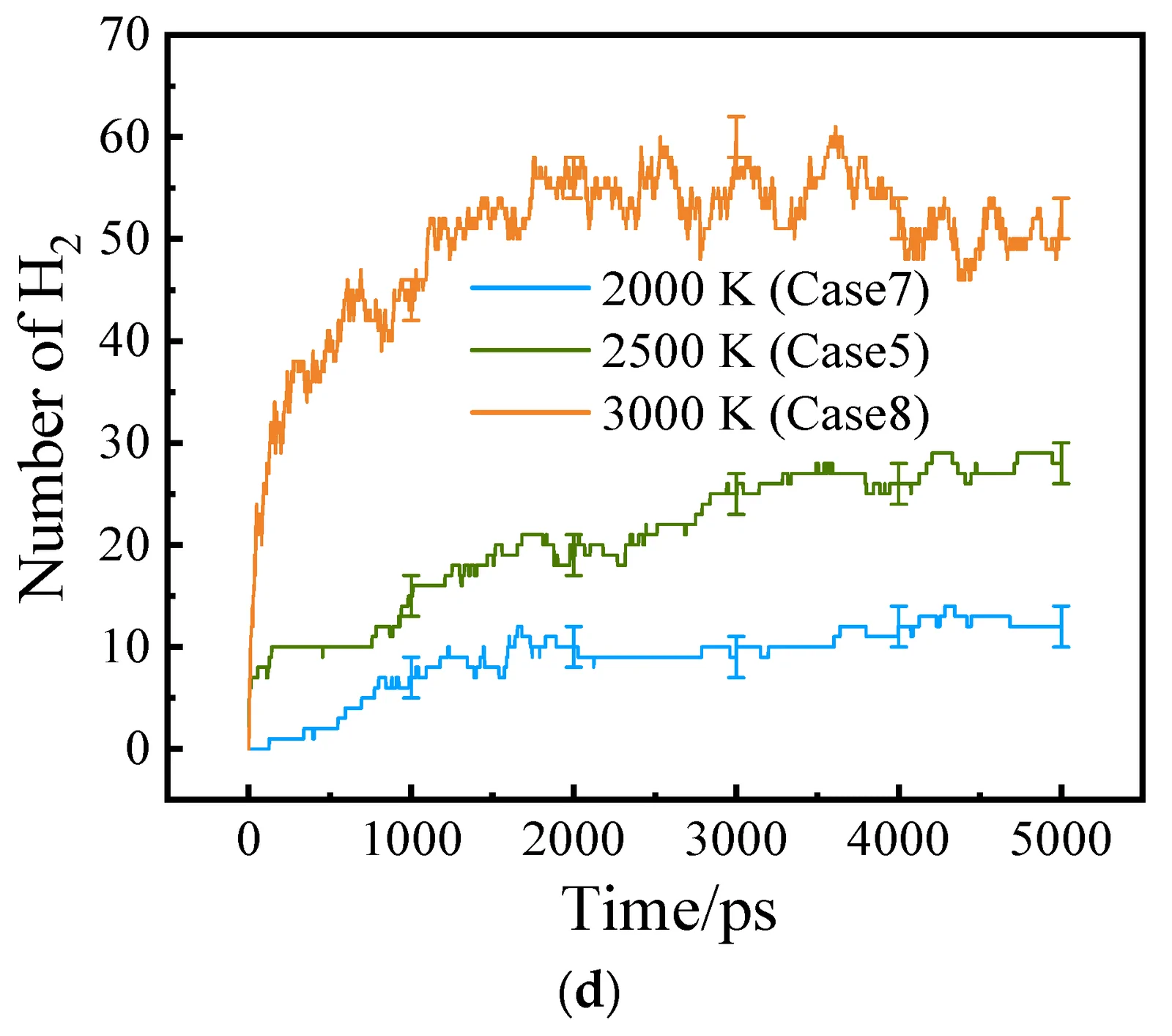
Effect of reaction temperature on the temporal evolution of species during C_2_H_4_ combustion with Fe addition: (**a**) C_2_H_4_, (**b**) O_2_, (**c**) H_2_O, (**d**) H_2_. Error bars denote mean ± 1 SD over three independent simulations.

The evolution of C_2_H_4_, O_2_, H_2_O and H_2_ with reaction time during the production of Fe-containing soot in C_2_H_4_ combustion at different reaction temperatures is given in Figure 4a–d, respectively. As a fuel in the combustion reaction process, C_2_H_4_ provides the necessary carbon source for soot generation. From Figure 4a, it is found that C_2_H_4_ is consumed rapidly and then slowly with the reaction until the fuel is completely pyrolyzed. As the reaction temperature increases from 2000 K to 2500 K and then further increases to 3000 K, the count of C_2_H_4_ molecules in the ensemble is 82 ± 2, 14 ± 2, and 1 ± 1 at 2 ns, respectively, and the consumption ratios of C_2_H_4_ are 31.7%, 88.3%, and 99.2%, respectively. The reaction rate of the fuel is accelerated. The oxidant is a necessary condition for the combustion process. It is found from Figure 4b that O_2_ is consumed rapidly and then slowly with the reaction until it is completely consumed, which is basically consistent with the evolution of C_2_H_4_. As the reaction temperature increases, the count of O_2_ molecules in the ensemble is 58 ± 2, 11 ± 2, and 0 at 2 ns, respectively. The consumption ratios of O_2_ are 35.6%, 87.8%, and 100%, respectively. The consumption ratio of O_2_ is also accelerated, which is highly consistent with the consumption ratio of C_2_H_4_. It can be observed that the consumption of fuel and oxidant is almost simultaneous in the initial stage of Fe-containing soot formation.

Figure 4c,d show the evolution of H_2_O and H_2_ with reaction time at different reaction temperatures. It can be seen from Figure 4c that H_2_O increases rapidly and then slowly with the reaction, especially in high temperature environment. As the reaction temperature increases from 2000 K to 3000 K, 39 ± 3, 118 ± 2, and 139 ± 4 H_2_O molecules are formed at 2 ns, respectively. It can be observed that the production rate of H_2_O is affected by temperature. This is mainly due to the accelerated consumption of C_2_H_4_ and O_2_ at high temperature, which provides a large count of H, O and OH and continuously reacts to form H_2_O. In Figure 4d, the evolution trend of H_2_ is largely consistent with that of H_2_O, and the production rate of H_2_ also accelerates with increasing reaction temperature. However, the overall abundance of H_2_ in the initial stage of soot formation is lower than that of H_2_O. Regarding the H_2_ formation mechanism, it is generally recognized that H_2_ in hydrocarbon pyrolysis and combustion is predominantly produced through the recombination of H atoms (H + H + M→H_2_ + M) and through hydrogen-abstraction pathways during fuel decomposition. Two candidate mechanisms may account for the lower abundance of H_2_ relative to H_2_O observed in the initial stage of soot formation: (i) the competitive consumption of H atoms by oxygen-containing radicals (e.g., OH + H→H_2_O, O + H_2_→OH + H), which diverts H atoms away from H_2_ formation; and (ii) the direct oxidation of H_2_ by abundant radical species (e.g., H_2_ + OH→H_2_O + H). We note that, within the present simulation framework, the relative contributions of these two channels have not been quantitatively deconvoluted; the above explanation is therefore presented as a hypothesis to be examined in future dedicated analyses rather than as a fully demonstrated reaction mechanism. Therefore, during the early stage of Fe-containing soot formation, elevated reaction temperatures accelerate the consumption of fuel and oxidizer, as well as the production of H_2_ and H_2_O.

### 2.3. Effect of Different Fe Loading Levels on Soot Formation During C_2_H_4_ Combustion

#### 2.3.1. Effect of Fe Loading Level on Soot Formation

**Figure 5:**
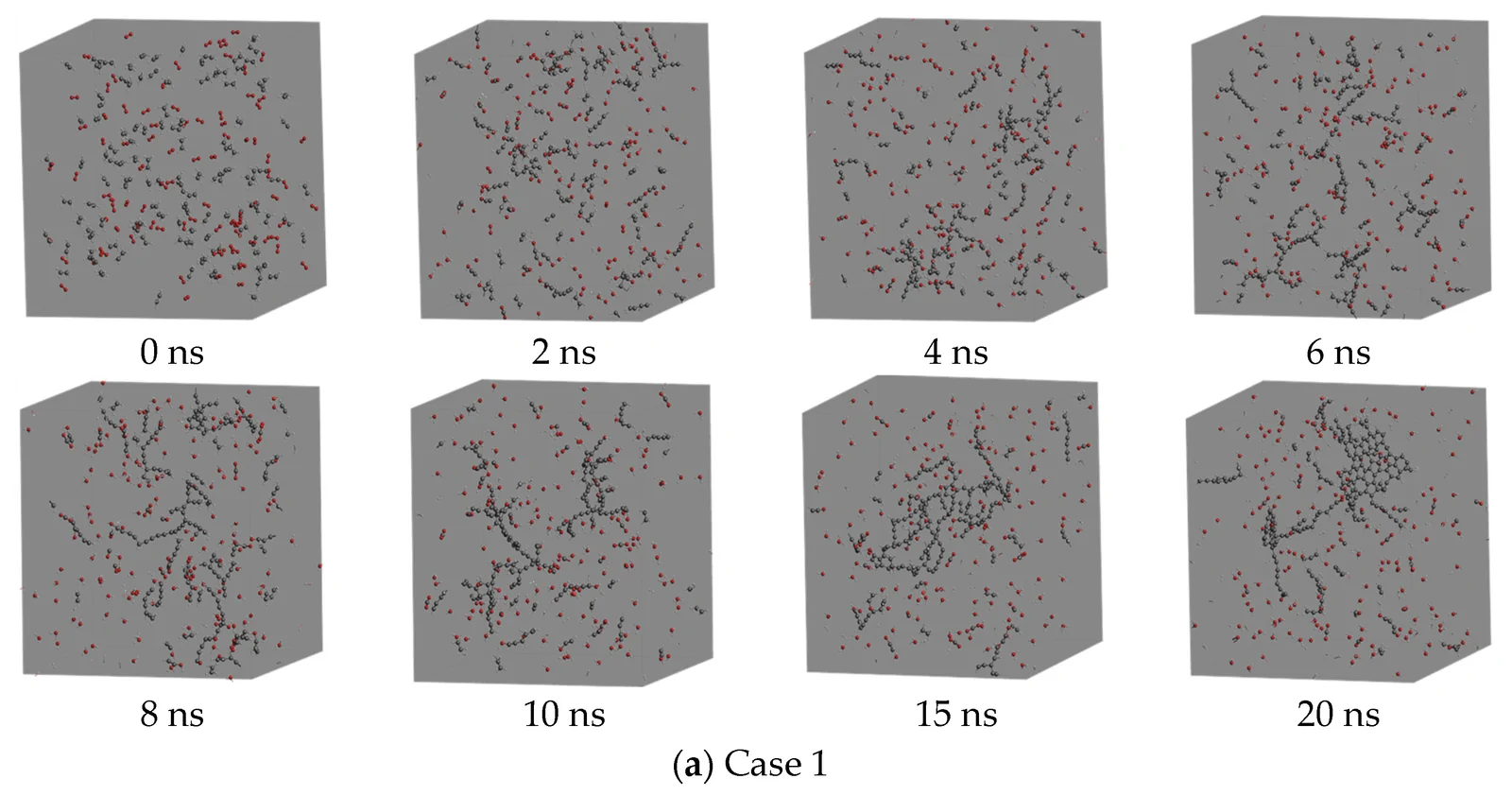

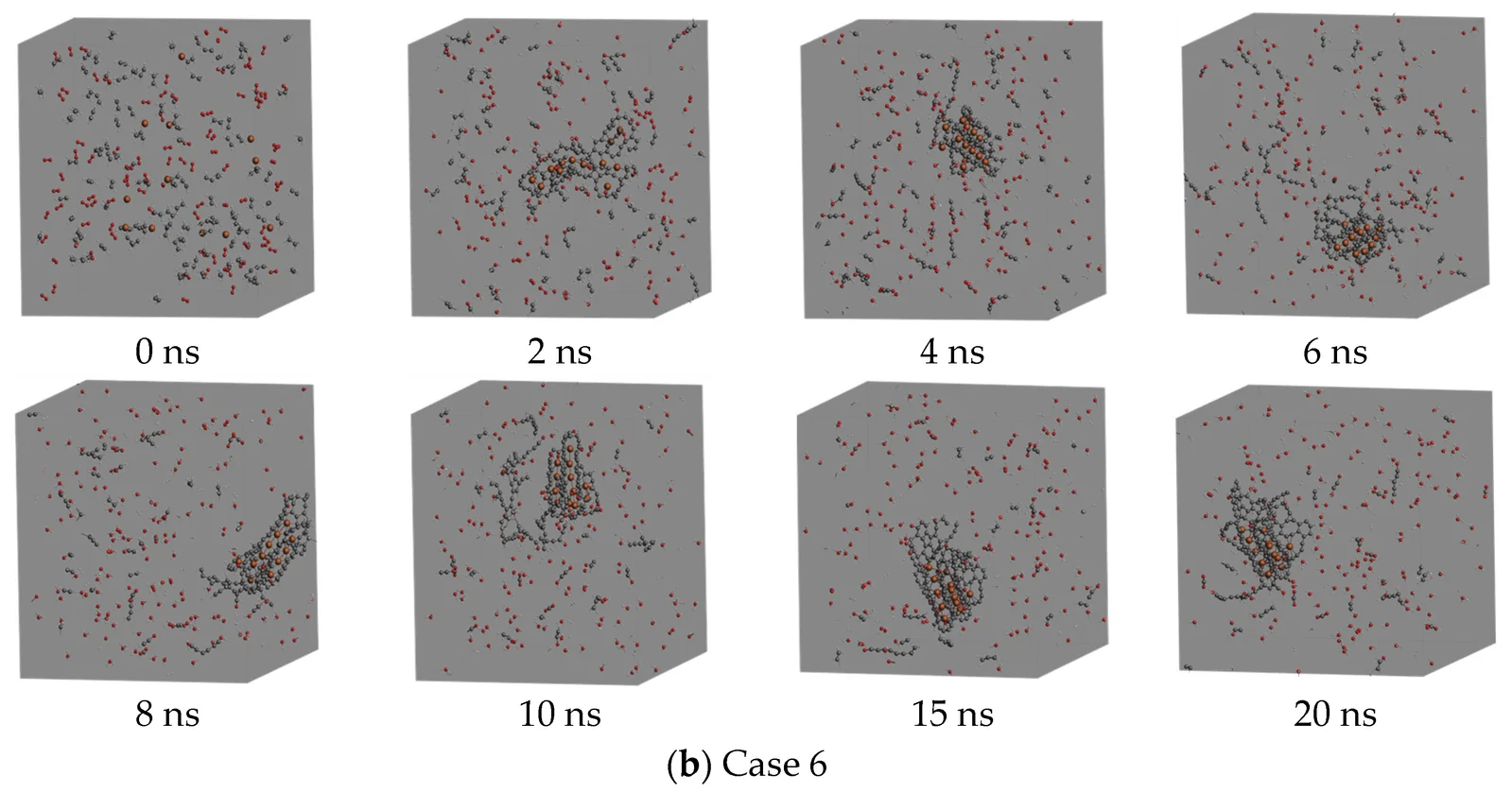
Effect of Fe addition on soot formation during C_2_H_4_ combustion: (**a**) Pure C_2_H_4_; (**b**) C_2_H_4_ with Fe addition.

Figure 5a,b presents the temporal evolution of PAHs or soot during pure C_2_H_4_ combustion and C_2_H_4_ combustion with Fe addition at the same reaction temperature (2500 K). As shown in Figure 5a, from the onset of the reaction to 2 ns, pure C_2_H_4_ undergoes continuous pyrolysis, forming a large count of short carbon chains. By 4 ns, the count of short carbon chains within the ensemble increases further, accompanied by the appearance of some longer carbon chains. At 6 ns, the population of long carbon chains continues to grow. By 10 ns, these carbon chains gradually polymerize to form two PAH molecules. As the reaction proceeds to 15 ns, a single nascent soot particle is formed within the ensemble. This nascent soot particle undergoes continuous growth and develops into a young soot particle by 20 ns, with both the carbon number and the count of six-membered rings within the particle increasing notably. In Figure 5b, we find that when the Fe-doped combustion is carried out at the same temperature, the reaction starts only at 2 ns, and an Fe-containing PAH with an Fe atom as the core is formed in the ensemble, and Fe-containing primary soot particles are formed at 4 ns. Subsequently, the Fe-containing primary soot particles continued to grow until 8 ns, which had grown into young Fe-containing soot particles with a more regular structure, but fewer internal ring structures. As the reaction progressed to 15 ns, the soot particles further evolved into mature Fe-containing soot particles, with a large count of five-membered ring and six-membered ring structures inside, and began to enter the graphitization stage until the simulation was terminated.

**Figure 6:**
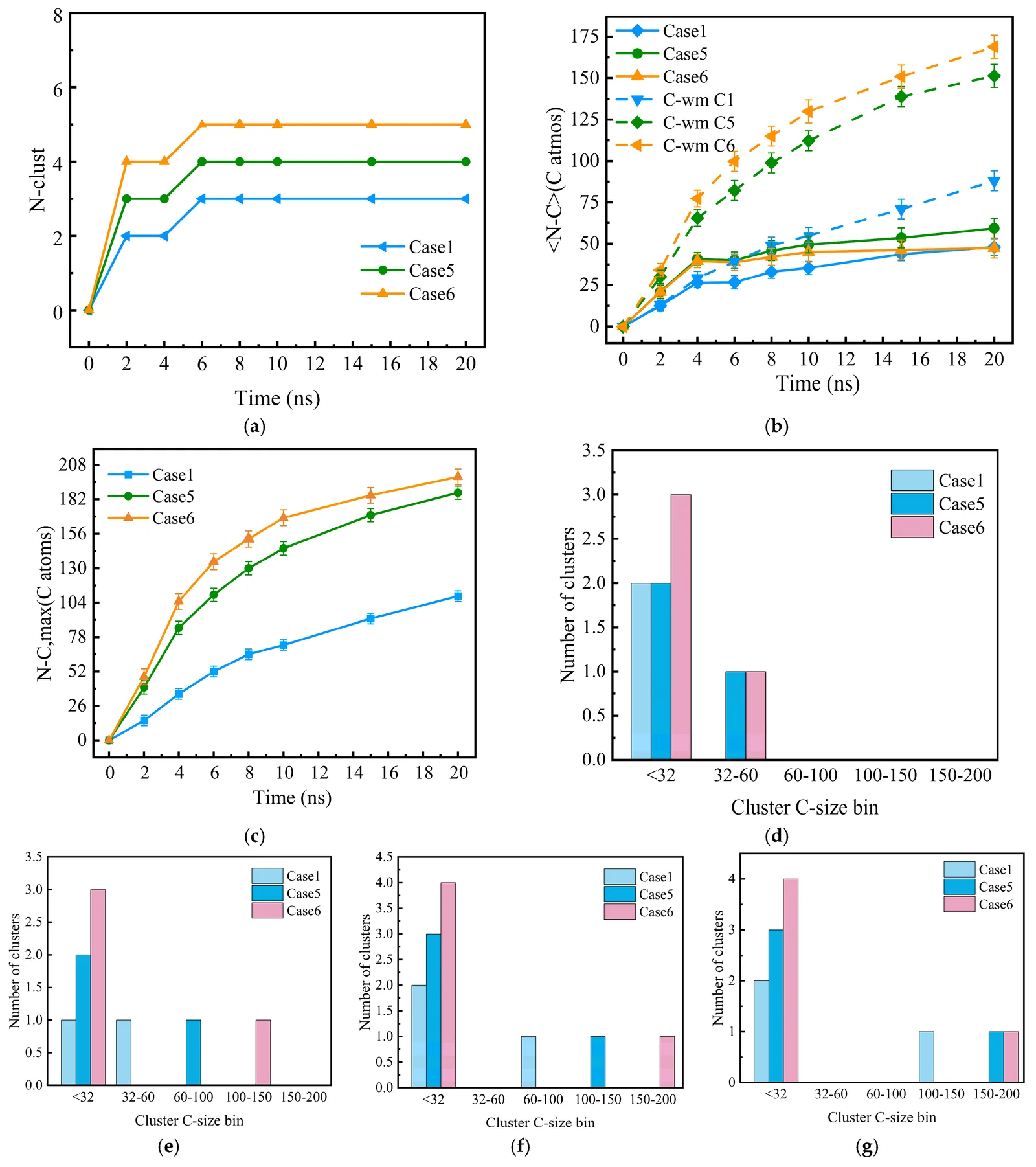
Soot population statistics during C_2_H_4_ combustion at 2500 K with different Fe loadings (Cases 1, 5, and 6). (**a**) Number of soot-like clusters (N_clust) as a function of time; (**b**) average cluster size (solid) and carbon-weighted mean cluster size (dashed) as functions of time; (**c**) largest-cluster carbon number as a function of time; (**d**–**g**) cluster-size distributions at 2, 4, 10, and 20 ns.

To verify that the promoting effect of Fe loading holds for the entire cluster population rather than only for the largest aggregate, we evaluated the population statistics shown in Figure 6. At 2500 K, the number of soot-like clusters (N_clust) increases from 2 to 3 to 4 at 2 ns and from 3 to 4 to 5 at 20 ns for Cases 1, 5, and 6, respectively. At 20 ns, the total carbon contained in all soot-like clusters is 144, 237, and 237 C atoms for Cases 1, 5, and 6 (out of 240 total C atoms in the system), with the remaining carbon residing in gas-phase species. The arithmetic mean cluster size is 48.0, 59.3, and 47.4 C atoms, respectively; the non-monotonic trend arises because higher Fe loading promotes continuous nucleation of new small clusters (N_clust increases from 3 to 5), which lowers the arithmetic average. In contrast, the carbon-weighted mean cluster size (ΣN_C^2^/ΣN_C) increases monotonically from 88.0 to 151.3 to 168.9 C atoms, indicating that an increasing fraction of the total carbon inventory is locked into the largest cluster as the Fe loading rises. The carbon-weighted mean is therefore the more appropriate descriptor for assessing Fe-promoted soot growth. Carbon-inventory conservation is satisfied in all cases (N_clust × arithmetic mean ≤ 240). The time-resolved cluster-size distributions show that, with increasing Fe loading, the distribution progressively shifts toward larger carbon-size bins at 4, 10, and 20 ns, whereas at 2 ns the clusters are predominantly small. These population-level statistics demonstrate that the promoting effect of Fe doping is systematic across the entire cluster population and is not an artifact of the largest-aggregate analysis, thereby strengthening the generality of the Fe-induced enhancement reported in Figure 6.

**Figure 7:**
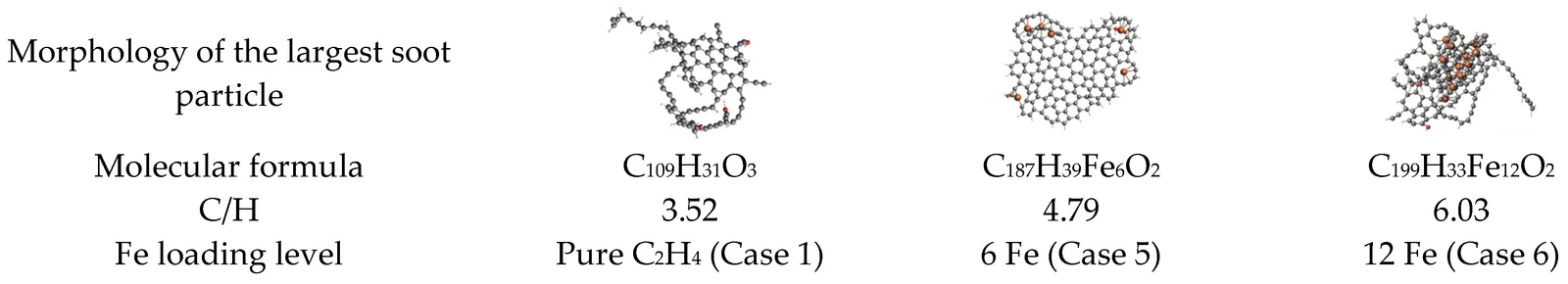
Morphology of the largest soot particles during C_2_H_4_ combustion at different Fe loading levels.

Figure 7 further shows the morphology and elemental composition of the largest detected soot particle obtained at 20 ns under C_2_H_4_ combustion with different Fe loading levels. Analysis based on this largest particle reflects the evolution of the biggest aggregate under each Fe loading condition and does not represent ensemble-averaged properties for all soot-like clusters within the simulation system. It can be found that the largest soot particles generated by pure C_2_H_4_ combustion at a reaction temperature of 2500 K within 20 ns are relatively small in shape and loose in structure. The interior contains 109 carbon atoms, which formally exceeds the mature-soot threshold (>100 C atoms). However, this cluster is at an early stage of maturation: its C/H ratio (3.52) and aromaticity index are substantially lower than those of the Fe-containing cases (C/H = 4.79 and 6.03), and its internal structure remains relatively loose with fewer fused aromatic rings. Thus, while Case 1 has reached the mature-soot classification by carbon number, its structural maturity is significantly lower than that of the Fe-doped cases. By mixing six Fe atoms in the C_2_H_4_ combustion process (Case 5), it was found that a compact mature Fe-containing soot particle was formed within 20 ns at the same reaction temperature. At this time, the soot contained 187 ± 5 carbon atoms and a large count of five-membered rings and benzene rings. When 12 Fe atoms are mixed in case 6, it can be found that a mature Fe-containing soot particle is also formed within 20 ns at the same reaction temperature, in which the Fe atoms act as nucleation cores around which the carbon skeleton develops, consistent with the measured reaction pathway in which Fe first forms Fe-C bonds and then Fe-containing carbon nuclei that coalesce into the soot particle (Table 2). The carbon-atom count of the soot is further increased to 199 ± 6, and the C/H is also increased from 4.79 to 6.03. In summary, increasing Fe loading is observed to promote the growth and maturity of the largest detected soot cluster under our tested conditions. We note that observations for this single largest cluster cannot be generalized to overall soot formation behavior of the entire ensemble. This trend for largest cluster evolution is consistent with the mechanism that Fe atoms promote PAHs activation and soot heterogeneous nucleation reported by Zhou et al. [12] and Mao et al. [13]. From a practical perspective, the Fe/C atomic ratios of 2.5% and 5.0% examined here are directly relevant to Fe–hydrocarbon co-combustion. They exceed the experimentally identified Fe/C threshold of ≈1.3%, at which Fe begins to homogeneously nucleate into nanoparticles in Fe-doped combustion [14], and are comparable to the molar doping levels typically used in ferrocene-added premixed ethylene flames [11]. Furthermore, the atomic (gas-phase) Fe adopted in the simulations represents the limiting case in which Fe is fully vaporized and atomically dispersed within the flame, as experimentally observed for Fe-doped flames [11]. Accordingly, although the present simulations address the atomistic regime, the promoting effect of Fe on soot nucleation and growth identified here is expected to be qualitatively transferable to practical Fe-hydrocarbon co-combustion systems.

#### 2.3.2. The Influence of Fe Content on the Evolution of Components

**Figure 8:**
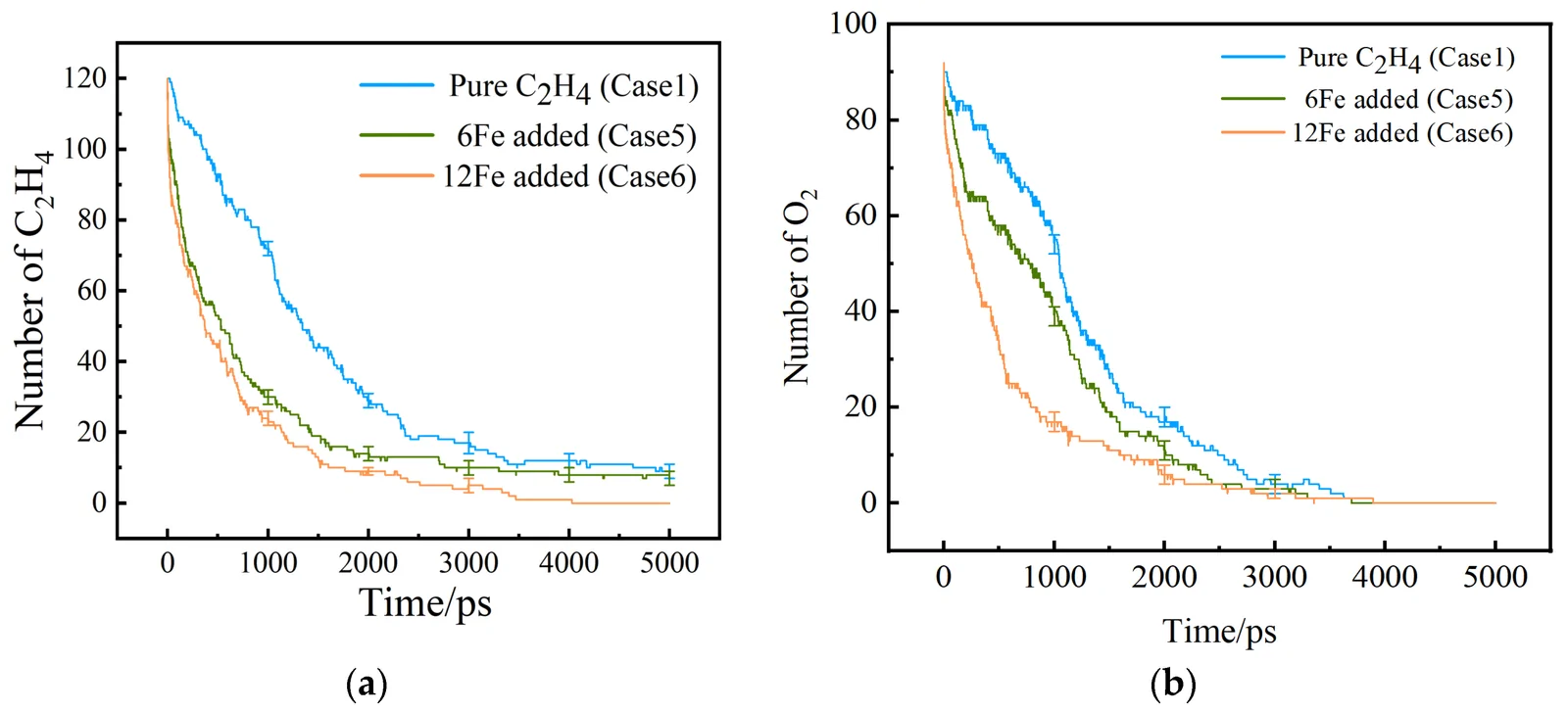

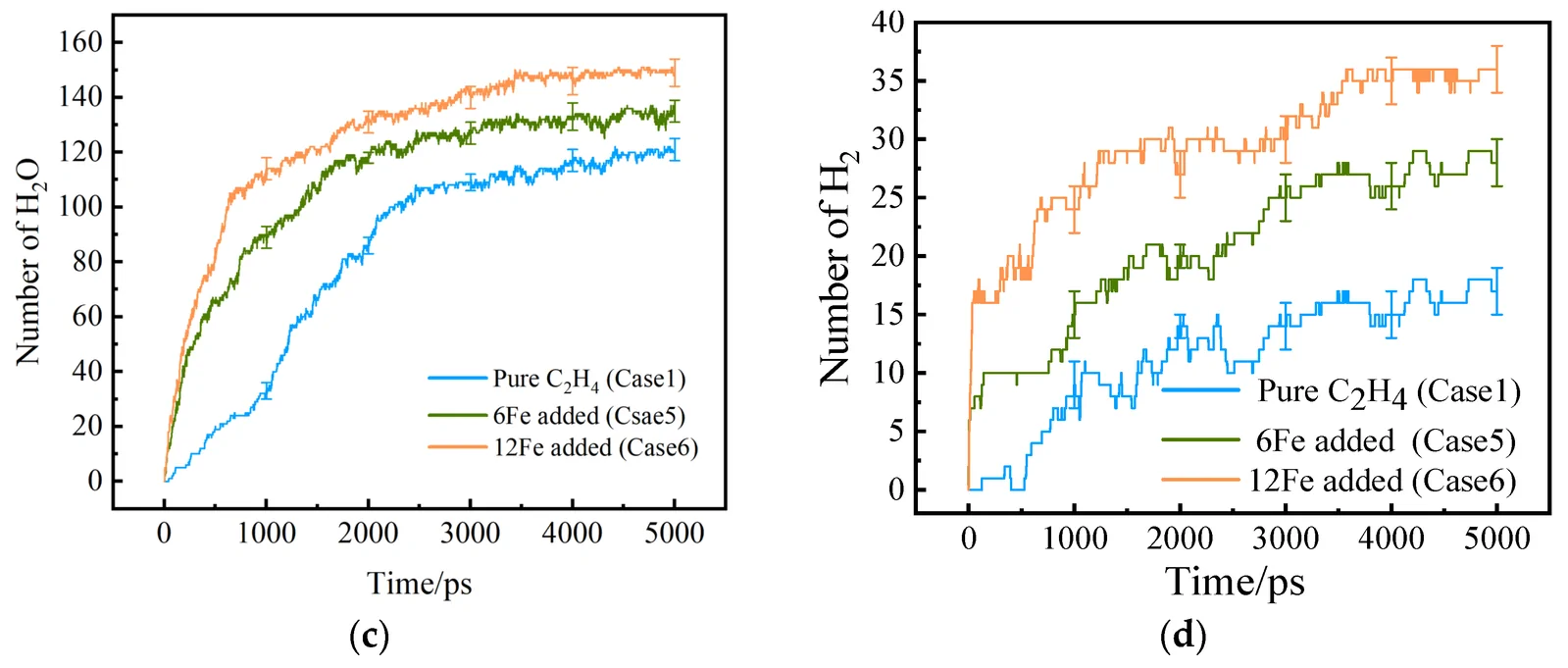
Effect of Fe addition on the temporal evolution of species during C_2_H_4_ combustion with Fe addition: (**a**) C_2_H_4_, (**b**) O_2_, (**c**) H_2_O, (**d**) H_2_. Error bars denote mean ± 1 SD over three independent simulations.

Figure 8a–d shows the evolution of C_2_H_4_, O_2_, H_2_O and H_2_ with reaction time during the production of soot in C_2_H_4_ combustion at the same reaction temperature and different Fe content. As shown in Figure 8a, increasing Fe loading accelerates the consumption rate of C_2_H_4_ within this system, although it does not alter the overall trend of rapid initial consumption followed by slower consumption. At 2 ns, the count of C_2_H_4_ molecules in Cases 1, 5, and 6 is 29 ± 2, 14 ± 2, and 9 ± 1, respectively, corresponding to consumption ratios of 75.8%, 88.3%, and 92.5%. However, for pure C_2_H_4_ and C_2_H_4_ with low Fe addition, a small fraction of C_2_H_4_ molecules remains unpyrolyzed at 5 ns. From Figure 8b, it is evident that increasing Fe loading continuously accelerates the consumption rate of O_2_. At 2 ns, the count of O_2_ molecules in the ensemble is 17 ± 2, 11 ± 2, and 6 ± 2 for Cases 1, 5, and 6, yielding consumption ratios of 81.1%, 87.8%, and 93.3%, respectively. Complete O_2_ consumption is achieved by 4 ns in all cases. As shown in Figure 8c,d, the evolution trends of H_2_O and H_2_ are generally consistent with each other, both exhibiting a rapid increase followed by a slower increase as the reaction proceeds. At 2 ns, the H_2_O counts for Cases 1, 5, and 6 are 86 ± 3, 118 ± 2, and 132 ± 4, while the H_2_ counts are 14 ± 1, 20 ± 1, and 27 ± 2, respectively. It can be observed that the rise in Fe content promoted the production of H_2_O and H_2_, and the production rate of H_2_O was much higher than that of H_2_, but the production rate of both products was greatly improved. In summary, under the present simulation conditions, increasing Fe loading accelerates the consumption rate of C_2_H_4_, O_2_ and elevates the production of H_2_O, H_2_. These trends should be interpreted in light of the small oxidant variation across cases. Across Cases 1, 5, and 6, the C_2_H_4_/O_2_ ratio varies only from 120/90 to 120/92, so that the nominal equivalence ratio and the C/O ratio change by less than 2.2% in total. Given that this marginal oxidant variation is far smaller than the pronounced changes in fuel/oxidant consumption and soot yield, the observed promotion is attributed predominantly to the chemical effect of Fe doping rather than to the minor oxidant adjustment. This attribution is further supported by two observations. First, the magnitude of the Fe-induced changes far exceeds the oxidant variation: across the Fe-loading series, the O_2_ count changes by only 2.2%, whereas the C_2_H_4_ consumption rate at 2 ns changes by 16.7% (75.8% to 92.5%), the largest-cluster carbon number changes by 82.6% (109 to 199), and the carbon-weighted mean cluster size changes by 91.9% (88.0 to 168.9). Second, the reaction-frequency analysis (Table 2) shows that Fe-specific bond transformations ((1) and (2)) occur exclusively in the Fe-containing cases and never in the Fe-free control, providing direct chemical evidence of Fe participation in soot nucleation.

#### 2.3.3. Reaction Path Analysis of Soot Formation in C_2_H_4_-Fe Combustion

**Figure 9:**
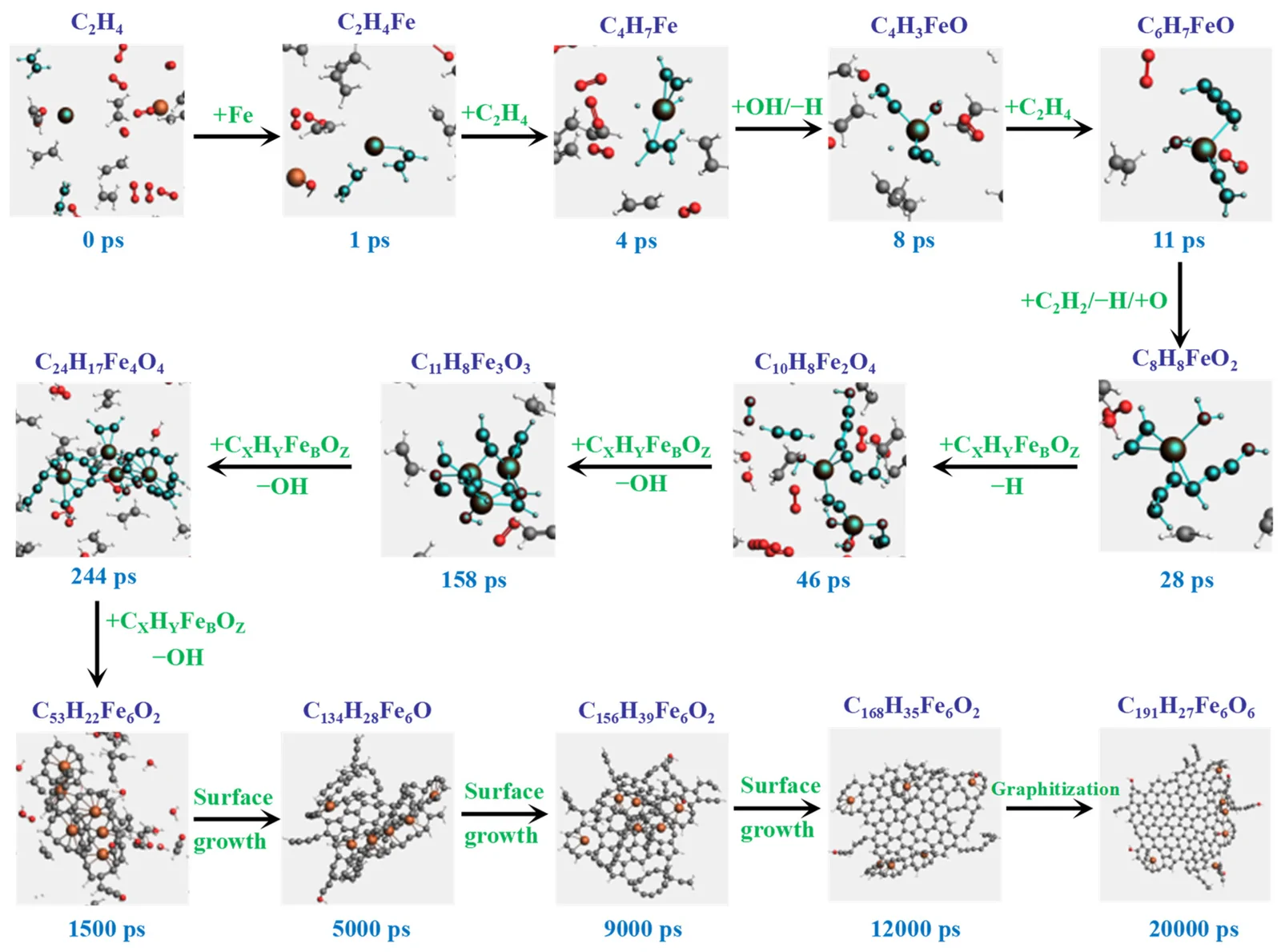
Typical pathways for the formation of PAHs and Fe-containing soot during C_2_H_4_ combustion with Fe addition.

In order to further analyze the effect of Fe doping on soot formation during ethylene combustion, this paper takes Case 8 as an example and combines Figure 9 to show the typical evolution path of PAHs and soot under ethylene Fe-doped combustion. Case 8 was selected as the representative case according to an objective criterion: among the three independent NVT trajectories, its evolution most closely matches the ensemble-averaged behavior, in particular with respect to the time to the first Fe-containing soot particle and the growth trajectory of the largest cluster. In addition, the complete reaction sequence from Fe addition to mature soot formation is reproduced across all three independent trajectories, confirming that the pathway shown in Figure 9 is typical rather than specific to a single run. The reaction path of this group is a representative molecular snapshot intercepted from the simulation trajectory, which can directly reflect the whole process of the evolution of Fe-containing PAHs to soot particles from the molecular level. It can be seen from Figure 9 that 120 C_2_H_4_ molecules and 6 isolated Fe atoms are distributed in the system at the initial time (0 ps) of the reaction. After the start-up of the reaction, the Fe atom preferentially binds to ethylene to initiate the carbon nucleus formation process, as shown in Equations (1) and (2):

(1) $$\begin{array}{c} \mathrm{Fe}+C_{2}H_{4}\to C_{2}H_{4}\mathrm{Fe} \end{array}$$

(2) $$\begin{array}{c} C_{2}H_{4}\mathrm{Fe}+C_{2}H_{4}\to C_{4}H_{7}\mathrm{Fe}+H \end{array}$$

In this work, an “Fe-containing carbon nucleus” is operationally defined as a molecular cluster that simultaneously satisfies three criteria: (i) it contains at least one Fe atom; (ii) it comprises a minimum number of carbon atoms—here taken as 8, corresponding to the first Fe-containing carbon core C_8_H_8_FeO_2_ identified in the trajectory; and (iii) it persists for a continuous period of at least 200 ps without complete dissociation, so that it can act as a seed for subsequent coalescence and surface growth. Clusters not meeting all three criteria are regarded as transient intermediates rather than nuclei. This criterion is applied consistently throughout the reaction-path and reaction-frequency analyses. The generated C_4_H_7_Fe undergoes a dehydrogenation reaction by OH attack in the system to form C_4_H_3_FeO (3) with free active sites. The active site can continue to capture ethylene molecules for an additional reaction (4), followed by dehydrogenation and acetylene addition at 28 ps, which gradually generates a single Fe small carbon core C_8_H_8_FeO_2_, as shown in Formula (5):

(3) $$\begin{array}{c} C_{4}H_{7}\mathrm{Fe}+\mathrm{OH}\to C_{4}H_{3}\mathrm{FeO}+5H \end{array}$$

(4) $$\begin{array}{c} C_{4}H_{3}\mathrm{FeO}+C_{2}H_{4}\to C_{6}H_{7}\mathrm{FeO} \end{array}$$

(5) $$\begin{array}{c} C_{6}H_{7}\mathrm{FeO}+C_{2}H_{2}+O\to C_{8}H_{8}\mathrm{Fe}O_{2}+H \end{array}$$

At this time, a number of similar small carbon nuclei have been formed in the whole system. In the next 18 ps, C_8_H_8_FeO_2_ merges with another small Fe-containing carbon nucleus and undergoes a dehydrogenation reaction to form C_10_H_8_Fe_2_O_4_ containing two Fe atoms. In the subsequent 112 ps, this small carbon nucleus containing two Fe atoms merges with another small carbon nucleus containing Fe, and a dehydroxylation reaction occurs to form a small carbon nucleus containing three Fe atoms—C_11_H_8_Fe_3_O_3_. After about 100 ps, the three-Fe-atom small carbon nucleus reacts with the single-Fe atom small carbon nucleus formed in the aggregate to form a larger carbon nucleus-C_24_H_17_Fe_4_O_4_. At 1500 ps, C_24_H_17_Fe_4_O_4_ merges with the small carbon nucleus containing the last two Fe atoms in the aggregate to form a new Fe-containing soot particle-C_53_H_22_Fe_6_O_2_.

After the formation of primary Fe-containing soot particles, the system enters the soot surface growth stage. The small molecules of ethylene and acetylene in the gas phase continue to undergo addition reactions on the surface of the particles, and the number of carbon atoms in the particles increases. In the evolution time of about 10,000 ps, the total number of carbon atoms increases to approximately 3.2 times the original, as shown in Equation (6):

C_53_H_22_Fe_6_O_2_ → [+C_2_H_4_/C_2_H_2_ surface addition, -H] → C_168_H_35_Fe_6_O_2_ (6)

In the late stage, graphitization is quantified by the aromaticity index (AI = N_ar/N_C × 100%), which increases from ~58% for the young soot to ~83% for the mature, more ordered soot, together with the C/H ratio and the development of regular five- and six-membered ring structures. As the C/H ratio increases, the internal skeleton of the carbon rearranges, and the Fe-containing soot particles C_191_H_27_Fe_6_O_6_ (7) with an ordered graphite structure are gradually formed. In the last 8000 ps, the number of carbon atoms only increased by 23, but the C/H ratio increased significantly from 4.8 to 7.1. It should be further noted that the arrows in (1)–(7) denote the observed net forward direction of the corresponding bond transformations extracted from the ReaxFF MD trajectories; they do not imply that the reactions are reversible or that chemical equilibrium has been established within the simulation time.

(7) $$\begin{array}{c} C_{168}H_{35}{\mathrm{Fe}}_{6}O_{2}\;\overset{\mathrm{graphitization}}{\to }\;C_{191}H_{27}{\mathrm{Fe}}_{6}O_{6} \end{array}$$

The reaction-frequency analysis over the three independent trajectories (Table 2) confirms that (1)–(7) are reproduced in every trajectory and in all Fe-containing cases (Cases 5, 6, 7, and 8), whereas they do not occur in the Fe-free control (Case 1). Early Fe-C_2_H_4_ association (1) and coalescence-driven surface growth (6) are the most frequent events, while single-Fe-nucleus formation (5) and graphitization (7) occur once per trajectory as rate-limiting steps. In summary, within the tested conditions, Fe doping promotes soot formation, especially in the stages of PAHs formation and soot nucleation. Reaction-path analysis shows that Fe atoms continuously combine with C_2_H_4_ and C_2_H_2_ molecules to generate various Fe-containing small carbon nuclei, which further develop into Fe-containing primary soot particles. The reaction frequencies in Table 2 were obtained by analyzing the molecular graphs at 1 ps intervals throughout the 20 ns production trajectory (20,000 frames per trajectory). A reaction event (1)–(7) was counted when the corresponding bond-order change exceeded the 0.3 cutoff and persisted for at least 100 fs (10 consecutive frames), excluding transient non-reactive collisions. The reported values are the mean and standard deviation over three independent NVT trajectories for Case 8. The complete (1)→(7) sequence was observed in all Fe-containing cases (5, 6, 7, 8) and in all three trajectories per case, confirming reproducibility. (1)–(7) represent the net forward bond transformations extracted from the trajectories; they are not conventional balanced elementary reactions, and the arrows do not imply reversibility or equilibrium.

## 3. Materials and Methods

A ReaxFF reaction force field molecular dynamics simulation, combined with the advantages of molecular dynamics and reaction force field, can accurately characterize the chemical reaction behavior of complex systems. The potential function is constructed based on the density functional theory, and the accuracy is approximate to that of quantum chemical calculation. Without presetting the reaction path, the microscopic characteristics of the system can be accurately quantified by parameters such as bond order, atomic structure, bonding characteristics and particle interaction [29]. Accordingly, as shown in Equation (8), the total energy of the system can be calculated by summing up the components of all energy terms:

*E_system_* = *E_bond_* + *E_over_* + *E_under_* + *E_val_* + *E_pen_* + *E_tors_* + *E_conj_* + *E_vdwaals_* + *E_coulomb_* (8)

The parameter definitions, molecular configuration, and applicability of the ReaxFF force field have been comprehensively detailed in prior work [23,30,31,32].

Table 3 lists all ReaxFF MD simulation cases for soot formation during C_2_H_4_ combustion with Fe powder addition in this study, where the system density (*ρ*), equivalence ratio (*φ*), and simulation time were set to 0.1 g/cm^3^, 4, and 20 ns, respectively. The *φ*, defined as *φ* = (3·N_C_2_H_4_ + 0.75·N_Fe)/N_O_2_, was nominally fixed at 4 for all cases. When Fe was added, the O_2_ molecule count was marginally increased (from 90 to 91 and 92) as a partial compensation for the oxygen consumed by Fe oxidation; this adjustment changes the nominal C/O ratio by less than 2.2% across the Fe-loading series, so that the oxidant environment is kept essentially constant and the observed differences can be attributed primarily to Fe doping. Given the current lack of available experimental data for C_2_H_4_ combustion with Fe powder addition, the Fe-containing force fields CHOFe-2012.ff and CHOFe-2015.ff were validated against the experimentally verified Fe-free force fields CHO-2016.ff and HE2.ff to select the appropriate reactive force field. Cases 1 through 4 correspond to the simulations of pure C_2_H_4_ combustion soot formation using the aforementioned four different reactive force fields; Cases 5 and 6 examine the influence of varying Fe loading levels on soot formation at 2500 K; and Cases 7 and 8 further investigate the influence of different reaction temperatures (2000 K and 3000 K) on soot formation during C_2_H_4_ combustion with Fe addition. Since the temperature and system density in ReaxFF MD simulations are held constant, the effects of Fe addition on soot formation at a given temperature are primarily manifested as chemical effects.

To facilitate comparison with practical Fe–hydrocarbon co-combustion systems and with available experimental data, the Fe doping levels were expressed by the normalized Fe/C atomic ratio. In Cases 5 and 6, 6 and 12 Fe atoms were introduced into a system containing 120 C_2_H_4_ molecules (240 C atoms), corresponding to Fe/C atomic ratios of 2.5% and 5.0% (Fe mass fractions of approximately 5.1% and 9.6% relative to the total system, respectively). The O_2_ molecule count was correspondingly increased from 90 to 91 and 92 so as to account for the oxygen consumed by Fe oxidation, thereby maintaining a consistent overall redox state across cases. These doping levels were selected because they bracket the experimentally reported threshold Fe/C ratio of ≈1.3%, above which homogeneously nucleated Fe-containing nanoparticles begin to form in Fe-doped diesel combustion [14], and because they fall within the range of metal-additive dosing applied in practical fuel applications. The lower level (2.5%) represents a near-threshold dopant concentration, whereas the higher level (5.0%) was chosen to amplify the Fe-promoting effect so that the underlying mechanism could be resolved more clearly, while keeping the Fe atoms sufficiently dilute and atomically dispersed, consistent with the atomic-state Fe observed in Fe-doped flames [11].

All cases in Table 3 are based on the ReaxFF module of Amsterdam Modeling Suite (AMS 2021.107) [33,34]. The three independent initial configurations were generated by randomly placing the C_2_H_4_, O_2_, and Fe molecules/atoms within the cubic simulation box. Cubic boxes with periodic boundary conditions in all three directions were used; the box side length was derived from the target density (0.1 g·cm^−3^) and the system composition, yielding L = 47.0, 47.9, and 48.8 Å for Cases 1, 5 (7, 8), and 6, respectively. Molecules were placed randomly and uniformly with a minimum intermolecular separation of ~2.0 Å. Three distinct random seeds were employed to generate the three configurations, ensuring independent initial molecular arrangements while keeping the ensemble composition, density, and temperature identical. Initial velocities were sampled from a Maxwell-Boltzmann distribution at the target temperature, and each system was started directly at that temperature. Systems were equilibrated for ~100 ps until the temperature fluctuation was within ±5% and the potential energy fluctuation within ~1 kcal/mol. Reactive MD was performed in the NVT ensemble using the Berendsen thermostat with a 0.1 fs integration time step [35]. This thermostat was selected for the following reasons. (i) It provides stable and computationally efficient temperature regulation and is widely adopted in ReaxFF-based combustion and pyrolysis studies, including the soot-formation simulations referenced in this work. (ii) In the strongly exothermic combustion process, the weak-coupling scheme smoothly dissipates the heat released by the reactions, thereby preventing artificial local temperature spikes during soot nucleation and growth, which is important for maintaining a well-defined temperature condition. (iii) Because all cases in this study were simulated under the identical thermostat setting, any systematic influence of the thermostat on reaction kinetics is common to all simulations; consequently, the relative (comparative) trends between different temperatures and Fe loadings—which constitute the main conclusions of this work—remain valid. It should nevertheless be noted that the Berendsen thermostat continuously drives the system toward the target temperature and does not reproduce the exact canonical (NVT) energy fluctuations. The simulations therefore represent near-isothermal conditions, and the temperature-dependent conclusions should be interpreted in this sense. Furthermore, the MD integration time step (0.1 fs) and the thermostat coupling time constant (100 fs) are distinct parameters; the former determines the numerical integration of the equations of motion, whereas the latter governs the rate at which velocities are rescaled toward the target temperature.

**Figure 10:**
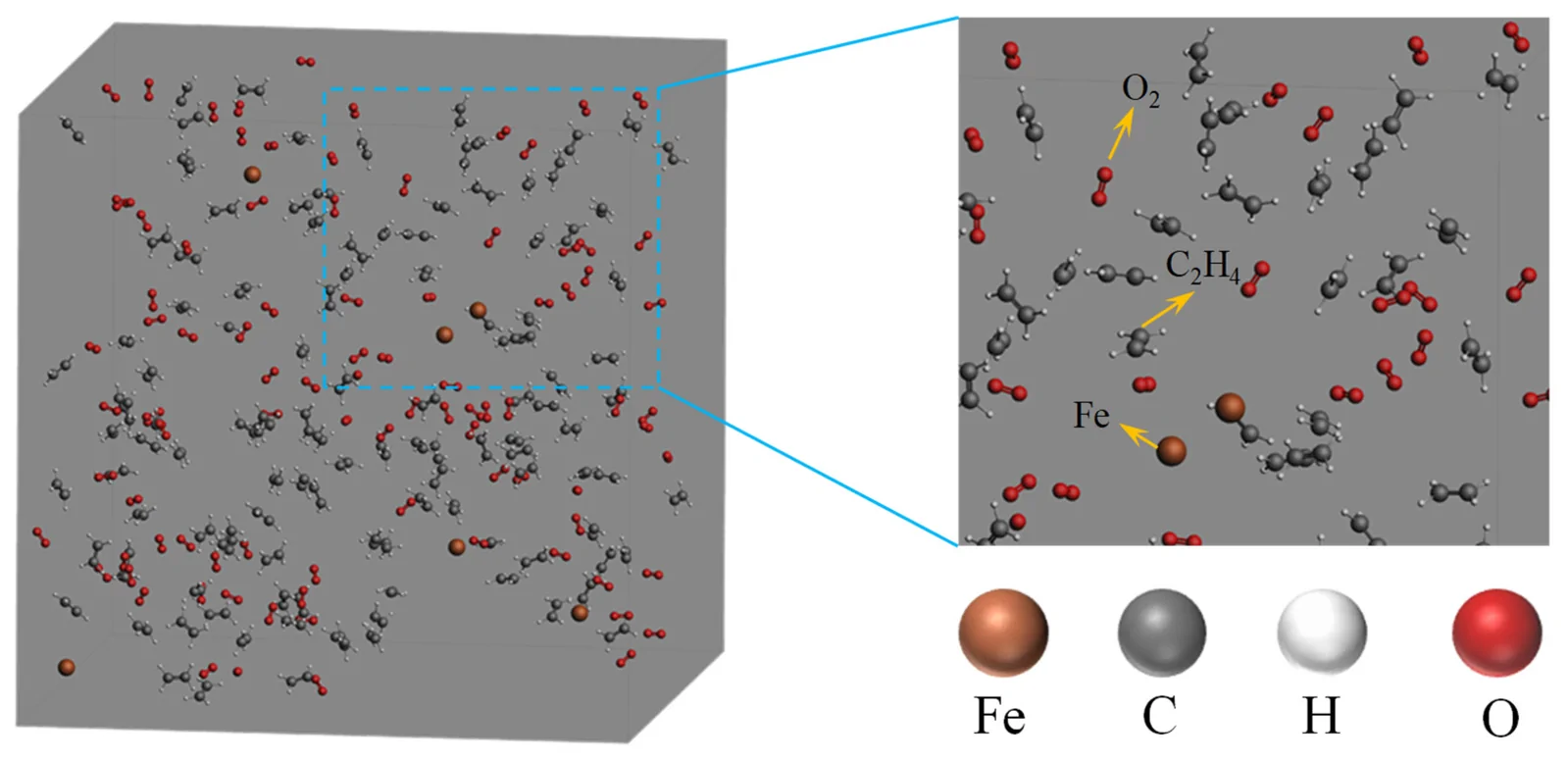
Optimized ensemble for C_2_H_4_ combustion with Fe addition.

Results were averaged over three independent NVT simulations with different initial configurations. Prior to the production runs, the energy and configuration of each system were pre-optimized using the geometry-optimization and energy-optimization modules of the simulation software, with the calculations converged to the default convergence criteria of the software (energy and force tolerances as implemented in the ReaxFF module). These default criteria ensure that the initial configurations are relaxed to a local energy minimum before the NVT production runs. Figure 10 is the optimized C_2_H_4_ Fe-doped combustion (Case 5) ensemble. Each case was simulated for 20 ns in the production phase, following an equilibration period of ~100 ps. Results were averaged over three independent NVT simulations (0.1 fs integration time step). On a single Xeon 8352Y node (64 cores, 256 GB memory, Intel Corporation, Santa Clara, CA, USA), the production run cost ~38 h per nanosecond of simulation time; one 20 ns trajectory therefore required ~760 h, and a full case (three trajectories) ~2280 h. All structural descriptors were computed from the molecular graphs extracted from the ReaxFF MD trajectories with a bond-order cutoff of 0.3. For each soot cluster, the total number of carbon atoms, N_C, was first determined. Ring structures were identified by a shortest-path ring-detection algorithm operating on the carbon–carbon bond-connectivity network. A ring was classified as aromatic when all of its carbon members were sp2-hybridized and the ring was conjugated (i.e., each C-C bond in the ring exhibited a bond order ≥ 1.5, characteristic of benzene-like rings); otherwise, the ring was classified as aliphatic (e.g., cyclopentadiene-like). On this basis, the aromaticity index (AI) and the aliphatic carbon fraction (F_al) were defined as:

(9) $$\begin{array}{c} \mathrm{AI}=(N\text{\_}\mathrm{ar}/N\text{\_}C)\times 100\% \end{array}$$

(10) $$\begin{array}{c} F\text{\_}\mathrm{al}=N\text{\_}\mathrm{al}/N\text{\_}C \end{array}$$

where N_ar is the number of carbon atoms located within aromatic ring structures, and N_al is the number of carbon atoms belonging to aliphatic moieties, i.e., non-aromatic carbons not incorporated into any ring (including short-chain and saturated carbons). The C/H ratio of a cluster was defined as the ratio of the total carbon-atom number to the total hydrogen-atom number within that cluster. On the basis of the total carbon-atom number N_C, soot particles were classified as nascent (N_C < 32), young (32 ≤ N_C ≤ 100), and mature (N_C > 100) soot, following the criteria adopted in previous ReaxFF-based soot studies and used consistently throughout this work. In addition to the largest-cluster descriptors, the overall soot population was evaluated. A “soot-like cluster” is defined as a carbon-containing cluster identified from the molecular graphs at each time frame using the bond-order cutoff of 0.3. The number of soot-like clusters (N_clust), the average cluster size (N_C), the carbon-weighted mean cluster size (ΣN_C^2^/ΣN_C), and the cluster-size distribution (number of clusters in each carbon-size bin) were computed at representative times (2, 4, 10, and 20 ns) and as functions of time. These statistics supplement the largest-cluster analysis and reflect the response of the entire cluster population.

As far as the ReaxFF MD method is concerned, its reliability and effectiveness have been fully verified and widely used by previous studies [23,25,36,37,38]. The authors [25] used CHO-2016.ff [39] force field and pyrolysis-gas chromatography/mass spectrometry to explore the chemical effects of CH_4_ and C_2_H_4_ combustion with H_2_ addition. The experimental and computational results show that the evolution of PAHs and initial soot particles, along with the influence of various chemical effects arising from hydrogen doping on their formation, has been clearly reflected. Kwon et al. [23] used the CHO-2016.ff force field to predict the soot formation ability of various hydrocarbon fuels, and the predicted values were consistent with the measured values of N_2_ diluted methane/air coflow flame. Wang et al. [38] employed the HE2.ff force field [40] together with the extinction method to investigate the soot formation characteristics and mechanisms during butane combustion with hydrogen and ammonia addition. Both experimental and simulation results revealed that i-butane exhibits a higher sooting tendency than n-butane, and that the addition of hydrogen and ammonia effectively suppresses soot formation during butane combustion. These findings confirm that the CHO-2016.ff and HE2.ff force fields exhibit high accuracy in characterizing pure fuel combustion without Fe addition, and can therefore serve as reliable benchmarks for validating the Fe-containing force fields CHOFe-2012.ff and CHOFe-2015.ff.

## 4. Conclusions

In this article, the formation characteristics of PAHs and soot particles in the combustion process of C_2_H_4_ doped with Fe were studied by ReaxFF MD method. The key catalytic role of Fe atoms in the nucleation and growth stages of soot was revealed, and the synergistic effect of reaction temperature and Fe content on soot maturity was clarified. It should be noted that the analyses of soot maturity, carbon-atom number, C/H ratio, temperature and other parameters in this study are all performed based on the largest soot cluster identified under each simulation condition. Accordingly, the relevant conclusions are only valid for clusters up to the maximum soot cluster detected in the simulated system. From this, the following main conclusions can be drawn:

(1) The reaction temperature has a decisive influence on the maturity of Fe-containing soot particles. At low temperature (2000 K), soot nucleation is slow, and only young soot particles with loose structure are formed at the end of the simulation. With the rise in temperature to 2500 K and 3000 K, the count of carbon atoms, C/H ratio and ring structure regularity in soot particles increased, and the Fe atoms gradually migrated from the inside to the edge of the particles, indicating that the heating not only accelerated the fuel pyrolysis and nucleation process, but also promoted the graphitization process of soot.
(2) Increasing Fe loading accelerates the growth and maturity of the largest soot cluster formed in C_2_H_4_-Fe co-combustion simulations. At the same temperature, the formation time of soot particles is obviously advanced after the addition of Fe atoms, and both the carbon atom number and the C/H ratio within the soot particles increase with higher Fe loading levels. At the same time, the presence of Fe accelerates the consumption of fuel and oxidant, and increases the production rate of H_2_O and H_2_, and the promotion effect increases with the rise in Fe content.
(3) The reaction path analysis shows that the Fe atom is the key to soot nucleation: the Fe atom first combines with C_2_H_4_ to form Fe-containing small molecules, then forms an Fe-containing carbon nucleus and develops into primary soot particles through dehydrogenation, addition and aggregation, and then forms mature particles through surface growth and graphitization. The mechanism of Fe promoting soot formation is clarified at the molecular level, which provides theoretical support for the research and development of clean combustion technology of Fe powder-hydrocarbon dual fuel.

It is important to distinguish the molecular-scale findings of this study from macroscopic soot-emission measurements. The present simulations demonstrate that Fe promotes PAH activation and accelerates soot nucleation and early growth under the tested high-temperature conditions. This finding pertains to the soot-formation (nucleation and early-growth) regime at the molecular scale and should not be directly extrapolated to net macroscopic soot emissions, which are additionally governed by soot oxidation, combustion efficiency, and aftertreatment/catalytic processes that are not captured within the present simulation time and system size. The apparent contrast between the enhanced molecular-scale nucleation reported here and the reduced engine-out soot emissions observed in engine experiments arises because the two findings concern different stages and spatial scales of the soot lifecycle: Fe intensifies the early formation stage resolved by ReaxFF MD, whereas the net emission level is further regulated by oxidation and removal in the later stages. Accordingly, the following limitations should be borne in mind when interpreting this work: (i) the finite simulation time (20 ns) and system size (≈120 fuel molecules) resolve only the early stage of soot evolution; (ii) the conclusions depend on the CHOFe-2012.ff force field, which is intended for qualitative mechanism analysis rather than quantitative prediction; (iii) the quantitative descriptors are based on the largest detected soot cluster and, although supplemented by population statistics, are not equivalent to full ensemble averages of the entire soot population; and (iv) the simulation temperatures (2000–3000 K) and the atomic (gas-phase) Fe representation differ from practical engine conditions. Within these limits, the qualitative mechanism of Fe-assisted soot nucleation identified here provides molecular-level insight, whereas quantitative prediction of macroscopic soot emissions remains beyond the scope of this work. (v) The force-field validation is indirect (based on comparison with other ReaxFF force fields and qualitative agreement with experimental trends), and direct quantitative experimental validation of Fe/C/H/O combustion kinetics remains a target for future work.

## Figures and Tables

**Table 1:** Comparison of peak concentrations of major combustion species during pure C_2_H_4_ combustion under different ReaxFF reactive force fields.

Combustion Species	Four Different ReaxFF Force Fields			
	CHOFe-2012.ff Case 1	CHOFe-2015.ff Case 2	CHO-2016.ff Case 3	HE2.ff Case 4
H_2_O	131	113	129	133
C_2_H_4_	120	120	120	120
O_2_	90	90	90	90
H_2_	45	58	44	42
C_2_H_2_	23	21	25	22
C_2_H_2_O	20	23	22	24
CO	19	47	17	21
C_2_H_3_	13	11	16	15
CO_2_	9	3	10	12
C_2_H_2_O_2_	7	9	11	9

**Table 2:** Reaction frequencies of key Fe-assisted soot-growth transformations in Case 8.

Reaction	Traj-1	Traj-2	Traj-3	Mean	Observed in Fe-Cases
1	6	5	7	6	Case 5, 6, 7, 8
2	4	4	5	4.3	Case 5, 6, 7, 8
3	3	3	2	2.7	Case 5, 6, 7, 8
4	2	3	3	2.7	Case 5, 6, 7, 8
5	1	1	1	1	Case 5, 6, 7, 8
6	8	9	8	8.3	Case 5, 6, 7, 8
7	1	1	1	1	Case 5, 6, 7, 8

**Table 3:** ReaxFF MD simulation cases for soot formation during C_2_H_4_ combustion with Fe powder addition.

Case	Fuel		Oxidant	*φ*	*T*/K	Force Field
	C_2_H_4_	Fe	O_2_			
1	120	0	90	4	2500	CHOFe-2012.ff
2	120	0	90	4	2500	CHOFe-2015.ff
3	120	0	90	4	2500	CHO-2016.ff
4	120	0	90	4	2500	HE2.ff
5	120	6	91	4	2500	CHOFe-2012.ff
6	120	12	92	4	2500	CHOFe-2012.ff
7	120	6	91	4	2000	CHOFe-2012.ff
8	120	6	91	4	3000	CHOFe-2012.ff

## Data Availability

The original contributions presented in this study are included in the article. Further inquiries can be directed to the corresponding authors.
